# Transcription-coupled structural dynamics of topologically associating domains regulate replication origin efficiency

**DOI:** 10.1186/s13059-021-02424-w

**Published:** 2021-07-12

**Authors:** Yongzheng Li, Boxin Xue, Mengling Zhang, Liwei Zhang, Yingping Hou, Yizhi Qin, Haizhen Long, Qian Peter Su, Yao Wang, Xiaodong Guan, Yanyan Jin, Yuan Cao, Guohong Li, Yujie Sun

**Affiliations:** 1 State Key Laboratory of Membrane Biology, Biomedical Pioneer Innovation Center (BIOPIC), School of Life Sciences, Peking University, Beijing, 100871 China; 2 Academy for Advanced Interdisciplinary Studies, Peking University, Beijing, 100871 China; 3 College of Chemistry and Molecular Engineering, Peking University, Beijing, 100871 China; 4 National Laboratory of Biomacromolecules, CAS Center for Excellence in Biomacromolecules, Institute of Biophysics, Chinese Academy of Sciences, Beijing, 100101 China; 5 Peking-Tsinghua Center for Life Sciences, Academy for Advanced Interdisciplinary Studies, Peking University, Beijing, 100871 China; 6 School of Biomedical Engineering, Faculty of Engineering and Information Technology, University of Technology Sydney, Sydney, NSW 2007 Australia; 7 Department of Neurobiology, Beijing Centre of Neural Regeneration and Repair, Capital Medical University, Beijing, 100101 China; 8 University of Chinese Academy of Sciences, Beijing, 100049 China; 9 College of Future Technology, Peking University, Beijing, 100871 China

**Keywords:** Replication origin, Topologically associating domain (TAD), Chromatin structure, Transcription, Super-resolution imaging, STORM

## Abstract

**Background:**

Metazoan cells only utilize a small subset of the potential DNA replication origins to duplicate the whole genome in each cell cycle. Origin choice is linked to cell growth, differentiation, and replication stress. Although various genetic and epigenetic signatures have been linked to the replication efficiency of origins, there is no consensus on how the selection of origins is determined.

**Results:**

We apply dual-color stochastic optical reconstruction microscopy (STORM) super-resolution imaging to map the spatial distribution of origins within individual topologically associating domains (TADs). We find that multiple replication origins initiate separately at the spatial boundary of a TAD at the beginning of the S phase. Intriguingly, while both high-efficiency and low-efficiency origins are distributed homogeneously in the TAD during the G1 phase, high-efficiency origins relocate to the TAD periphery before the S phase. Origin relocalization is dependent on both transcription and CTCF-mediated chromatin structure. Further, we observe that the replication machinery protein PCNA forms immobile clusters around TADs at the G1/S transition, explaining why origins at the TAD periphery are preferentially fired.

**Conclusion:**

Our work reveals a new origin selection mechanism that the replication efficiency of origins is determined by their physical distribution in the chromatin domain, which undergoes a transcription-dependent structural re-organization process. Our model explains the complex links between replication origin efficiency and many genetic and epigenetic signatures that mark active transcription. The coordination between DNA replication, transcription, and chromatin organization inside individual TADs also provides new insights into the biological functions of sub-domain chromatin structural dynamics.

**Supplementary Information:**

The online version contains supplementary material available at 10.1186/s13059-021-02424-w.

## Introduction

DNA replication is an exquisitely regulated process. Its deregulation may lead to genome instability and tumorigenesis [1]. In metazoans, duplication of the genome is initiated at tens of thousands of discrete chromosome loci known as replication origins. Intriguingly, while a mammalian cell has a total of ~ 250,000 potential replication origins, it only uses a small subset (~ 10%) to duplicate the whole genome [2–5]. It has been under debate whether the selection of origins is random or regulated. Single cell-based measurements, including the classic DNA combing assays [6–8] and the recent single-cell sequencing studies [9, 10], showed that cells rarely use the same cohort of origins to duplicate the genome. Nevertheless, both single cell and population-averaged origin mapping experiments have confirmed that not all origins are equal, and they rather have differential probabilities of firing, namely origin efficiency [4], against a random origin selection mechanism.

The mechanisms that determine the origin efficiency remain enigmatic. Various genetic and epigenetic signatures, including CpG islands, G-quadruplexes, nucleosome-depleted regions, and histone modifications, are found to correlate with origin efficiency, but a consensus principle is still lacking [2]. The origin efficiency has been also suggested to link with chromatin structures [4, 11, 12]. Several earlier studies have revealed a relationship between replicons and chromatin loops [7, 13–15]. Beyond the loop structure, more recent studies have shown that the spatiotemporal initiation of replication is regulated at the chromatin domain level. Genome-scale mapping of replication kinetics showed that DNA replication in metazoan cells takes place in a defined temporal order with the genome segmented into large chromosomal regions, known as replication domains (RDs) [4, 5]. Each RD contains multiple replicons with uniform and constant replication timing. Importantly, the boundaries of RDs are found to align precisely with that of topologically associating domains (TADs) [16]. TADs are physical compartmentalization units of the genome that are stable over multiple cell cycles and conserved across related species [17]. Thus, this finding provides strong supports for the correlation between DNA replication and the three-dimensional (3D) structure of chromosomes. A typical RD is about 1 Mb and contains a few dozens of potential origins. These origins do not have similar replication efficiencies as only several of them are actually used to replicate the domain [2–5]. In temporal space, direct measurements on spread-out DNA fibers by DNA combing experiments have shown that the high-efficiency origins which have a higher chance to be used within a RD fire nearly synchronously [6–8]. However, how the origin efficiency is spatially regulated in a RD has been an outstanding question.

In physical space, mapping of the spatial arrangements of replication sites by in situ fluorescence imaging in the nucleus showed that DNA replication initiates at thousands of discrete puncta termed replication foci (RFi) [7, 18–23]. Provided that the number of pulse-labeled RFi is much less than the number of high-efficiency origins [7, 18–20], RFi are thought to be the equivalents of RDs defined in temporal space and contain multiple replicons. Based on the collective evidence from the DNA halo [7, 13–15], DNA combing [6, 7, 24], and RFi imaging [7, 18–20], the Rosette model was proposed to illustrate the spatiotemporal organization and regulation of high-efficiency origins in a RD [4]. In this model, a RD contains multiple loops which form a rosette-like structure with the high-efficiency origins clustered and co-fired in the chromatin domain. The Rosette model is further supported by another study showing that depletion of cohesin, a protein complex scaffolding the rosette-like structure, reduces the number of origins used for genome duplication [25]. However, as previous imaging studies were mostly limited in their spatial resolution and lack of sequence-specific labeling of TADs and replication origins, there is no direct imaging evidence to support the clustered origin distribution. In a recent work, Cardoso and his colleagues applied SIM super-resolution imaging (resolution ~ 100 nm) and identified more replicons than conventional imaging (resolution ~ 300 nm) [21]. Importantly, with the improvement in resolution, nearly all replicons are found to be spatially separated at the beginning of the S phase, which casts doubt on the clustering of replication origins proposed in the Rosette model. In the accompanying work, they proposed a stochastic, proximity-induced (domino-like) replication initiation model, in which the high-efficiency origins are not necessarily clustered spatially in the domain but the domino-like replication progression leads to clustering of replicons [26].

A thorough understanding of how the physical structure of RDs regulates origin efficiency needs in situ imaging of the spatial distribution of both high-efficiency and low-efficiency origins within the TADs. Given that a TAD is about 800 kb [16] with a radius of gyration less than 300 nm [27] and contains a few dozens of potential origins, dual-color 3D super-resolution imaging with ultra-high resolution in all three dimensions is a pre-requisite to distinguish which origins are more preferentially used among the many potential candidates within individual TADs. Moreover, new labeling strategies are necessary for low-efficiency origins because the traditional approach based on metabolic pulse-labeling only labels high-efficiency origins. Here, we applied a recently developed chromatin painting and imaging technique, namely OligoSTORM [28, 29], to investigate whether origin efficiency is dependent on TAD structure. OligoSTORM combines Oligopaints [30] with stochastic optical reconstruction microscopy (STORM) [31]. Oligopaints are high-efficiency oligonucleotide fluorescent in situ hybridization (FISH) probes based on PCR strategy that can robustly label whole chromosomes or any specific chromosomal regions. STORM and its equivalents PALM/fPALM [32, 33] are single-molecule localization-based super-resolution imaging techniques that have the highest spatial resolution (~ 20 nm laterally and ~ 50 nm axially) among all super-resolution imaging methods [34]. With the best of both sides, OligoSTORM has been successfully applied to resolve the fine physical chromatin structures, such as TADs and compartments, in single cells [27, 35–37].

Using OligoSTORM, we performed, to our knowledge, the first quantitative characterization of TAD structural dynamics and the spatiotemporal distribution of replication origins within individual TADs in different cell cycle stages at sub-diffraction-limit resolution. We found that replication initiation generally takes place at the spatial boundary of a TAD. In the G1 phase, TADs undergo a transcription-dependent structural re-organization process, which exposes a subset of origins to the spatial boundary of the TAD. We also observed an interesting peri-RFi distribution of the major replication machinery protein PCNA, in line with the observation that replication initiation generally takes place at the spatial boundary of a TAD. Thus, our work reveals a new origin selection mechanism that the replication efficiency of origins is determined by their physical distribution in the chromatin domain and transcription plays a role in the chromatin structural re-organization. This new mechanism transcends the scope of specific genetic and epigenetic signatures for origin efficiency and also provides new insight into the biological functions of sub-domain chromatin structural dynamics.

## Results

### Replication origins initiate separately at the periphery of a TAD

In order to investigate the role of chromatin structure in origin selection, we chose to directly visualize how replication initiation is spatially organized and regulated within individual RDs using STORM imaging. Two RDs were chosen from the replication timing profile of HeLa cells (Additional file 1: Figure S1a). The boundaries of either RD are overlaid with that of a TAD, which are hereafter named as TAD1 and TAD2, respectively (Additional file 1: Figure S1b). TAD1 (Chr1:16911932-17714928) is an early replicating domain and enriched of transcriptionally actin histone modifications. TAD2 (Chr1: 17722716-18846245) is a middle replicating domain and enriched of transcriptionally repressed histone modifications (Additional file 1: Figure S1c). The two TADs were labeled by the Oligopaint approach using 12,000 TAMRA-modified primary oligonucleotide probes targeting the TADs and then imaged by STORM (the “Methods” section). Morphological characterization showed that the radii of gyration of TAD1 and TAD2 are about 200 nm (Additional file 1: Figure S2), which is consistent with previous work [27, 36]. Moreover, even though the genomic length of TAD2 is larger than that of TAD1, the physical size of TAD2 is significantly smaller than that of TAD1 (Additional file 1: Figure S2b), suggesting that TAD2 is more compact. This observation is consistent with the previously reported findings that active chromatin domains are more open than repressed chromatin domains [27, 36], thereby benchmarking the technical rigor of our TAD labeling and imaging.

**Fig. 1:**
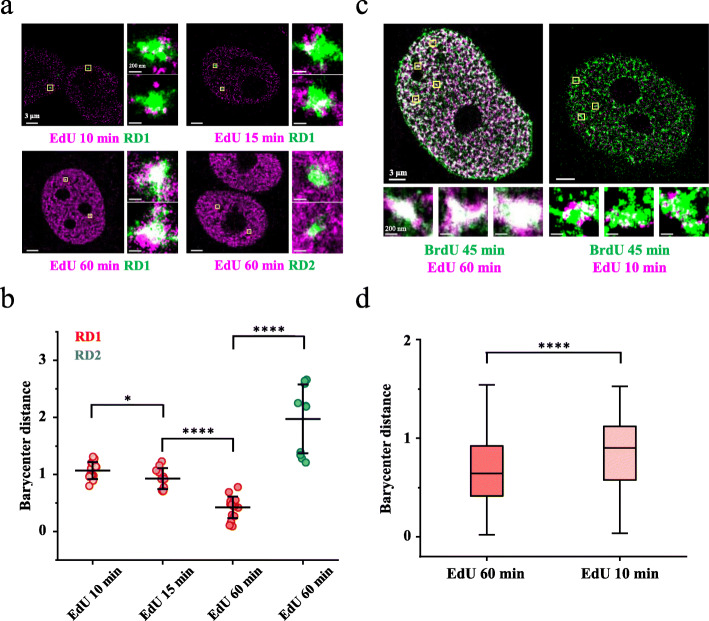
Super-resolution imaging of RFi and TADs in the S phase. **a** Representative STORM images of TAD1 and TAD2 labeled by Oligopaint probes (green) and RFi labeled metabolically for different durations (purple) (the “Methods” section). TAD1 and TAD2 were chosen based on the replication timing profile and Hi-C interaction heatmap of HeLa cells (Additional file 1: Figure S1). TAD1: an early replicating domain (Chr1:16911932-17714928). TAD2: a middle replicating domain (Chr1:17722716-18846245). Metabolic labeling of DNA replication was performed by supplying EdU to the cell upon release into the S phase for 10 min, 15 min, and 60 min (purple). The areas inside the yellow squares are shown at higher magnification to the right of each nucleus. Portions of the two signals that overlap are shown in white. **b** Barycenter distances between the TAD and its spatially associated RFi (the “Methods” section) in **a**. Horizontal lines and error bars represent the mean values ± s.d. of three or more independent biological replicates (n = 16 cells). **c** Representative STORM images of RFi labeled metabolically for different durations in two consecutive cell cycles. Consecutive metabolic labeling of DNA replication was performed by supplying BrdU (green) to the cell upon release into the S phase in the first cell cycle, followed by supplying EdU (purple) to the cell upon release into the S phase in the second cell cycle (for indicated durations). The areas inside the yellow squares are shown at higher magnification below each nucleus. **d** Box plot of barycenter distances between BrdU and EdU-labeled RFi in **c** (data were pooled from n = 10 cells). Center line, median; box limits, 25% and 75% of the entire population; whiskers, observations within 1.5× the interquartile range of the box limits. Significance was analyzed by an un-paired two sample parametric t test. ****P < 0.0001, ***P < 0.0005, **P < 0.01, *P < 0.05, N.S. not significant. 3D results are shown in Fig. S2&S5

Next, to visualize the replication initiation sites in the TADs, we took the classic metabolic pulse-labeling strategy [7, 23]. Briefly, we first synchronized HeLa cells to the boundary of the G1 phase and S phase as previously described [7, 38] (the “Methods” section). Immediately after release of the replication arrest at the G1/S boundary, we performed a 10-min pulse labeling of the replicating DNA by supplying thymidine analog EdU, which was then labeled with Alexa647 by click chemistry after fixation of the cell (the “Methods” section). This synchronization procedure can synchronize nearly 80% cells at the G1/S transition and minimally impacts the growth and morphology of cells [7, 23, 38] (Additional file 1: Figure S3). Following labeling and fixation, we applied dual-color STORM (the “Methods” section) to image the TADs (Fig. 1a, green) and the replication initiation sites, which appeared as punctate foci (Fig. 1a, purple). The punctate distribution of 10-min pulse-labeled foci imaged by STORM in our work was similar with those previously imaged by other groups with SIM [21].

We should note that the positions of these 10-min pulse-labeled foci can precisely represent the position of the corresponding replication initiation sites for two reasons. First, as EdU was added immediately after the release of replication arrest, the majority of the pulse-labeled RFi were supposed to contain the corresponding replication initiation sites. Second, the size of 10-min pulse-labeled foci (roughly 20 kb [7, 21, 23]) is ~ 30 nm in diameter, as revealed in the super-resolution images (Fig. 1a and Additional file 1: Figure S8c), which is close to the lateral resolution of STORM imaging. As the spatial resolution (directly related with the single-molecule localization precision) sets the minimal apparent size of a target imaged by STORM [31], there would be no difference in the apparent size or position when imaging a 20-kb genomic region and a much smaller sub-region, e.g., the replication initiation site in the region.

**Fig. 2:**
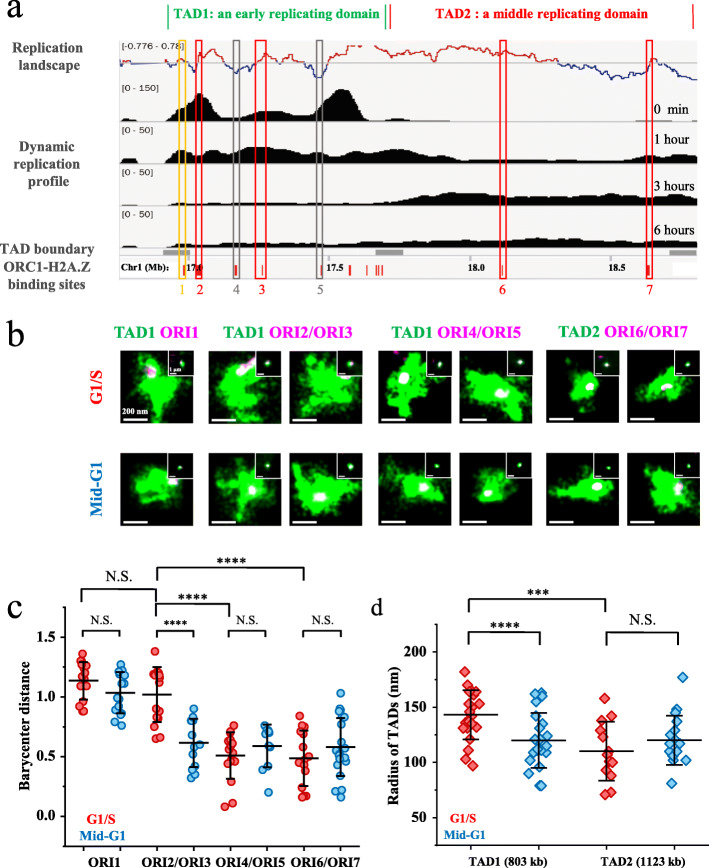
Spatial distribution of replication origins relative to the TADs in the G1 and G1/S phases. **a** A scheme of replication in TAD1 and TAD2. The top profile represents the replication landscape obtained by OK-seq. (−0.776–0.78) was the threshold of OK-seq [40]. The middle black peaks represent the dynamic replication profile, which was obtained by 10-min BrdU pulse labeling at 0 min, 30 min, 3 h, and 6 h into the S phase. (0–50) or (0–150) is the range of normalized BrdU-seq data. The gray bars represent the TAD boundaries in the RDs. The small red bars at the bottom represent the ORC1 and H2A.Z binding sites indicating the potential replication origins. Representative high-efficiency and low-efficiency replication origins defined by the BrdU-seq data and the OK-seq profile are marked with vertical rectangles. Yellow rectangle: high-efficiency replication origin (ORI1) at the TAD boundary. Red rectangles: high-efficiency replication origins in TAD1 (ORI2 and ORI3) and TAD2 (ORI6 and ORI7). Black rectangles: low-efficiency replication origins in TAD1 (ORI4 and ORI5). **b** Representative STORM images of TADs (green) and their origins (purple) labeled by FISH with oligoprobes in the G1 and G1/S phases. Upper, TADs and origins labeled at the G1/S transition. Lower, TADs and origins labeled at approximately 5 h into the G1 phase. Portions of the two signals that overlap are shown in white. The corresponding conventional images are shown in the inset. **c** Barycenter distances between origins and TADs in **b** (n ≥ 10 cells). To reduce the number of groups, the barycenter distance of each ORI was measured separately and displayed as four groups. **d** Radii of TAD1 and TAD2 in the G1 or G1/S phase (n ≥ 10 cells). For lines and statistics in **c** and **d**, see the description in the legend of Fig. 1. 3D results are shown in Fig. S11

Intriguingly, the replication initiation sites were found to preferentially localize at the physical boundary of the early replicating TAD1 as shown in the two insets in the upper left panel of Fig. 1a, which are close-up view of the two allelic TAD1 and their associated replication initiation sites. We applied SR-Tesseler [39], a recently developed robust and unbiased segmentation algorithm, to quantitatively analyze TADs and origins in super-resolution images (the “Methods” section and Additional file 1: Figure S4). To describe the relative spatial relationship, we defined barycenter distance as the physical distance between the barycenter of a TAD and the barycenter of RFi normalized by the radius of gyration of the TAD (Fig. 1b) (the “Methods” section). The barycenter is the mass density center of all single-molecule localizations in a TAD or RFi. For randomly distributed foci within the TAD, the expected barycenter distance is 0.71 (the “Methods” section). We measured the barycenter distances between the 10-min pulse-labeled RFi and TAD1 which were near 1 (Fig. 1b), indicating a peripheral distribution of the replication initiation sites in TAD1. To gauge the sensitivity of our method, we also measured the barycenter distances of 15-min pulse-labeled RFi, which were closer to the center of TAD1 in comparison with 10-min pulse-labeled RFi (Fig. 1b), showing that our method is highly sensitive as a means of detecting the spatial distribution of replication origins in a TAD. As a control, RFi labeled for 60 min starting at the G1/S boundary were well overlaid on TAD1 (Fig. 1a and Additional file 1: Figure S5) with barycenter distances near 0.5 (Fig. 1b and Additional file 1: Figure S5), but did not show obvious overlap with the middle replicating TAD2 (Fig. 1a, b), consistent with the fact that TAD2 begins to replicate at approximately 3 h into the S phase (Additional file 1: Figure S1 and Fig. 2a).

To further validate the analysis of spatial localization of replication initiation sites relative to the TAD, we also applied DBSCAN [41], a density-based spatial clustering algorithm, to extract individual RFi and quantify their spatial localization in TADs based on 2D and 3D STORM images (Additional file 1: Figure S5) (the “Methods” section). The spatial relationship of RFi relative to the TAD rendered by DBSCAN in 2D or 3D images (Additional file 1: Figure S5a-c) was identical with those obtained by the SR-Tesseler analysis (Fig. 1b). We also defined radial density distribution (RDD), which is the median radial distribution of all single-molecule detections of the RFi in a TAD normalized by the radius of the TAD, to quantify the spatial distribution of RFi in TADs in 3D images (the “Methods” section). A larger RDD value indicates a more peripheral distribution of RFi in a TAD. The spatial distribution of RFi revealed by RDD was similar to that obtained by the barycenter distance (Additional file 1: Figure S5c/d). The analyses described above cross-validated each other and eliminated the possibility of artifacts possibly introduced by foci identification, inter-foci distance measurement algorithms, or projection of 3D images onto the 2D plane. To investigate whether the above findings obtained with TAD1 are true for other early replicating TADs, we imaged another two early replicating TADs (TAD3 and TAD4) as well as a late replicating TAD (TAD5) as a control (Additional file 3: Table S2). The results of these TADs were consistent with that obtained on TAD1 and TAD2, respectively (Additional file 1: Figure S6). We note that cell cycle synchronization by aphidicolin treatment may cause replication stress. To eliminate the possibility that the aphidicolin treatment may artificially lead to our observations, we designed experiments to observe replication initiation sites at the early S phase without cell cycle synchronization (Additional file 1: Figure S7). We proved that replication initiation also takes place at the periphery of a TAD in non-synchronized cells (Additional file 1: Figure S6), consistent with the observation in aphidicolin-treated cells. Besides HeLa cells, we also checked other cell lines including hRPE, U2OS, and MDA-MB-231 and obtained the same results (Additional file 1: Figure S6). Therefore, these data together demonstrated that multiple replication origins initiate separately at the spatial periphery of early replicating TADs.

Next, to check whether the above findings obtained with several individual TADs are generally true for all early replicating TADs, a high-throughput labeling method is needed. Provided that the boundaries of RDs and TADs are precisely aligned [16], we took a metabolic labeling strategy to label all early RDs and their associated replication initiation sites by two rounds of BrdU and EdU pulse labeling at the beginning of the S phase over two consecutive cell cycles, respectively (Fig. 1c). EdU was labeled with Alexa647 by click chemistry, whereas BrdU was immunostained with atto-550 [7]. It has been suggested that RFi labeled by thymidine analogs EdU or BrdU for 1 h generally correspond to RDs [7], as replication of early RDs takes approximately 1 h [7, 19, 42, 43]. We confirmed this assumption by showing the large overlapping between FISH-labeled TAD1 and its corresponding 60-min EdU-labeled RFi (Fig. 1a lower left, Additional file 1: Figure S5 and Additional file 1: Figure S8a/b). Because the spatial density of 60-min metabolically labeled RFi was very high, which impeded the confidence in the algorithm with regard to identification of the boundaries between spatially adjacent RDs, we chose to label RDs using a 45-min labeling duration. As shown by the dual-color STORM imaging in Fig. 1c, early RDs double-labeled for 45 min and 60 min in two consecutive cell cycles merged very well (Fig. 1c). RFi labeled for 45 min were slightly smaller than those labeled for 60 min, albeit insignificant (Additional file 1: Figure S8c), supporting the usage of 45-min labeled RFi to represent early RDs. As a control experiment, we showed that the size increase from RFi labeled for 10 min to those labeled for 15 min was successfully detected (Additional file 1: Figure S8c), demonstrating the high detection sensitivity of STORM imaging and also excluding the possibility that the insignificant size difference between RFi labeled for 45 min and 60 min was due to insufficient detection sensitivity. We also checked whether different thymidine analogs, e.g., EdU and BrdU, introduced any difference in the size of RFi. The STORM images showed no significant difference between EdU- and BrdU-labeled RFi (Additional file 1: Figure S8d).

We then labeled all early RDs by 45-min BrdU pulse in the first cell cycle followed by labeling the replication initiation sites with 10-min EdU pulse in the second cell cycle (Fig. 1c right). We obtained a global view of the spatial distribution of replication initiation sites relative to early RDs in a cell. Analysis of the dual-color STORM images showed that there were averagely 7 replication initiation sites in each RD, in good agreement with previous estimations [5, 44] as well as the fact that the sizes of a RD and a replicon are about 800 kb [16] and 120 kb [6–8], respectively. These analyses thereby benchmarked the technical rigor of labeling and imaging of RDs and associated origins.

We next calculated the barycenter distances between replication initiation sites (EdU 10 min) and RDs (BrdU 45 min) and found that they were significantly larger than those between doubly labeled (EdU 60 min and BrdU 45 min) RDs (Fig. 1d). Lastly, a similar spatial pattern was observed when Cy3B or dUTP was used respectively instead of atto-550 or BrdU, thereby eliminating the possibility that the observed pattern could be the consequence of labeling or detection artifacts associated with specific dyes (Additional file 1: Figure S9). Taken together the data of both particular RDs and metabolically labeled RDs, we conclude that the fired replication origins in a RD are spatially separated (Fig. 1a, c and 7 replication initiation sites per domain), which is in direct contrary with the classic Rosette model [4] and in line with previous findings discovered by SIM imaging [21, 45] or TEM imaging [46]. More importantly, these spatially separated replication origins tend to initiate at the periphery of RDs, implicating a role of chromatin domain structure in regulating the efficiency of replication origins.

### High-efficiency origins relocate inside-out to the periphery of early replicating TADs in the G1 phase

Only 10–20% of the origins in a TAD are used for DNA replication during each cell cycle, while the rest stay dormant. Given the observation that replication tends to initiate at the periphery of an early replicating TAD (Fig. 1 and Additional file 1: Figure S5), we next imaged both high-efficiency and low-efficiency origins to check whether the spatial distribution of origins in a TAD is related to their replication efficiency. As low-efficiency origins cannot be fluorescently labeled by metabolic pulse labeling, OligoSTORM was applied to label and image the TADs and origins. To obtain the replication efficiency of origins in TAD1 and TAD2, we first measured the dynamic replication profile of HeLa cells using BrdU-seq [47] by 10-min BrdU labeling at 0 min, 1 h, 3 h, and 6 h into the S phase (Fig. 2a, black peaks). The BrdU-seq profile reveals that TAD1 replicates in the first hour of the S phase, while TAD2 starts to replicate after about 3 h, which is in line with the replication timing profile (Additional file 1: Figure S1). All potential replication origins in TAD1 and TAD2 were mapped by ORC1 and H2A.Z ChIP-seq [48] of HeLa cells. We aligned the dynamic replication profile and ORC1 binding sites with the previously reported replication landscape of the HeLa cell genome [40] (Fig. 2a). Based on the origin efficiency revealed by both the replication landscape and the dynamic replication profile, 3 representative high-efficiency origins (ORI1, ORI2, and ORI3, marked by yellow and red boxes) and 2 representative low-efficiency origins (ORI4 and ORI5, marked by black boxes) were chosen in TAD1. Two high-efficiency origins (ORI6 and ORI7, marked by red boxes) were chosen in TAD2 (Additional file 2: Table S1). We note that while aphidicolin is commonly used in the investigation of DNA replication [6–8], it is concerned that such replication stress may stimulate the engagement of low-efficiency origins in the activated replication domains [49]. In our study, to avoid selection of abnormally activated low-efficiency origins, we combined the dynamic replication profile measured by BrdU-seq under aphidicolin treatment and the replication landscape measured by OK-seq without aphidicolin treatment. With this strategy, we were able to select low-efficiency replication origins (ORI4 and ORI5), which were not affected by aphidicolin treatment, for FISH labeling in the TADs. For metabolic labeling of replication initiation sites with aphidicolin treatment, although some low-efficiency origins have the chance to be fired under the replication stress (Fig. 1), it does not affect the conclusion that replication starts at the periphery of the chromatin domains.

After choosing origins with different replication efficiency in TAD1 and TAD2, we then applied dual-color OligoSTORM to image the TADs and their associated origins at the G1/S transition (the “Methods” section). In order to ensure ample fluorescent signal for individual replication origins, Oligopaint probes were designed to target a ~ 20-kb genomic zone containing the replication origin (the “Methods” section). As discussed above, limited by spatial resolution, there would be no difference in the apparent size or position when imaging a 20-kb genomic region and a much smaller sub-region, e.g., the replication initiation site in the region. The results showed that at the G1/S transition, all 3 high-efficiency origins in TAD1 were located at the spatial periphery of TAD1 (Fig. 2b, upper) with large barycenter distances (Fig. 2c, red). In contrast, the low-efficiency origins (ORI4 and ORI5) were located at the interior of TAD1 (Fig. 2b, upper) with barycenter distances much shorter than those of ORI1, ORI2, and ORI3 (Fig. 2c, red). Interestingly, the high-efficiency origins in TAD2 (ORI6 and ORI7), which are not supposed to fire until the middle S phase, were found to locate inside of the domain at the G1/S transition (Fig. 2b, upper) with small barycenter distances (Fig. 2c, red). Taken together, these results suggest that the replication efficiency of origins at the G1/S transition is correlated with their physical positions in the TAD.

Eukaryotic DNA replication is tightly orchestrated with the cell cycle. In the canonical two-step activation model [4], licensing of origins occurs with pre-RC formation in the G1 phase followed by origin activation and initiation in the S phase. Recent Hi-C studies have shown that the structure of TADs changes from the G1 phase to the S phase [50]. We wondered how the spatial distribution of origins in a TAD changes accompanying the chromatin structure, which could serve as determinants of selective origin initiation. We thus imaged the TADs and origins in the mid-G1 phase (approximately 5 h post-G1 onset) (the “Methods” section), which is after the timing decision point (TDP) when the replication timing program becomes established and TADs reform [51]. Strikingly, we found that the high-efficiency origins ORI2 and ORI3 were located inside of TAD1 in the mid-G1 phase (Fig. 2b, lower) with small barycenter distances (Fig. 2c, blue), in sharp contrast to their peripheral localization in TAD1 at the G1/S transition (Fig. 2b, upper and Fig. 2c, red). On the contrary, low-efficiency origins ORI4 and ORI5 were found to remain inside of TAD1 from the mid-G1 (Fig. 2b, lower and Fig. 2c, blue) to the G1/S transition (Fig. 2b, upper and Fig. 2c, red). These observations suggested that high-efficiency origins undergo an inside-out relocation process in the TAD, possibly along with the chromatin structural re-organization within the TADs that occurs in the G1 phase. Interestingly, unlike ORI2 and ORI3, high-efficiency origin ORI1 did not relocate but remained at the TAD periphery from the mid-G1 phase to the G1/S transition (Fig. 2b, c). We note that, in the sequence space, ORI1 is at the insulation boundary of TAD1 (Fig. 2a and Additional file 2: Table S1), and therefore, structural re-organization within the TADs would not affect its peripheral localization relative to the TAD. Such correspondence between the sequence boundary and the physical boundary of a TAD was also reported in a recent study [36], thereby again benchmarking the technical rigor of labeling and imaging of TADs and associated origins.

To further investigate the relationship of replication origins and chromatin loops within the TADs, we aligned origins with the sites enriched of CTCF and cohesin genome wide (the “Methods” section) (Additional file 1: Figure S10). CTCF and cohesin are the key scaffold protein complexes bound at the anchor sites of the chromatin loops as well as the TAD boundary [52]. Compared with random DNA loci, CTCF-cohesin binding sites were generally enriched with replication origins. High-efficiency origins colocalized better with CTCF-cohesin binding sites than low-efficiency origins. In addition, origins located at TAD boundaries (the “Methods” section) were of higher replication efficiency than those located inside the TADs. These sequencing results again emphasized the relationship of replication efficiency with chromatin organization within the TADs.

In addition to the structural re-organization within the TADs, we found that the physical size of TAD1 also became larger at the G1/S transition in comparison with its size in the G1 phase (Fig. 2d), while this change was not detected for TAD2. Note that the volume increase was not the result of DNA replication, as the cells were arrested at the G1/S transition, suggesting that the chromatin of TAD1 undergoes de-compaction in the G1 phase, which is in line with the results of Hi-C analysis showing that intra-domain chromatin interactions decrease in the G1 phase [50]. Analysis of 3D STORM images led to the same findings (Additional file 1: Figure S11), which again eliminated the possibility of artifacts introduced by projection of 3D images onto the 2D plane. To further prove the findings on TAD1 and TAD2, we imaged another two early replicating TADs (TAD3 and TAD4) as well as a late replicating TAD (TAD5). The results of these TADs were consistent with that obtained on TAD1 and TAD2, respectively (Additional file 1: Figure S12). Taken together, these data revealed that the structural re-organization within the TADs and de-compaction in the G1 phase facilitate the relocation of high-efficiency origins from the TAD interior to the periphery, supporting the observation that DNA replication initiates at the periphery of TADs in the beginning of S phase (Fig. 1).

### Distinct spatial localization of high-efficiency and low-efficiency origins at the G1/S transition is correlated with chromatin loops and dependent on transcription

**Fig. 3:**
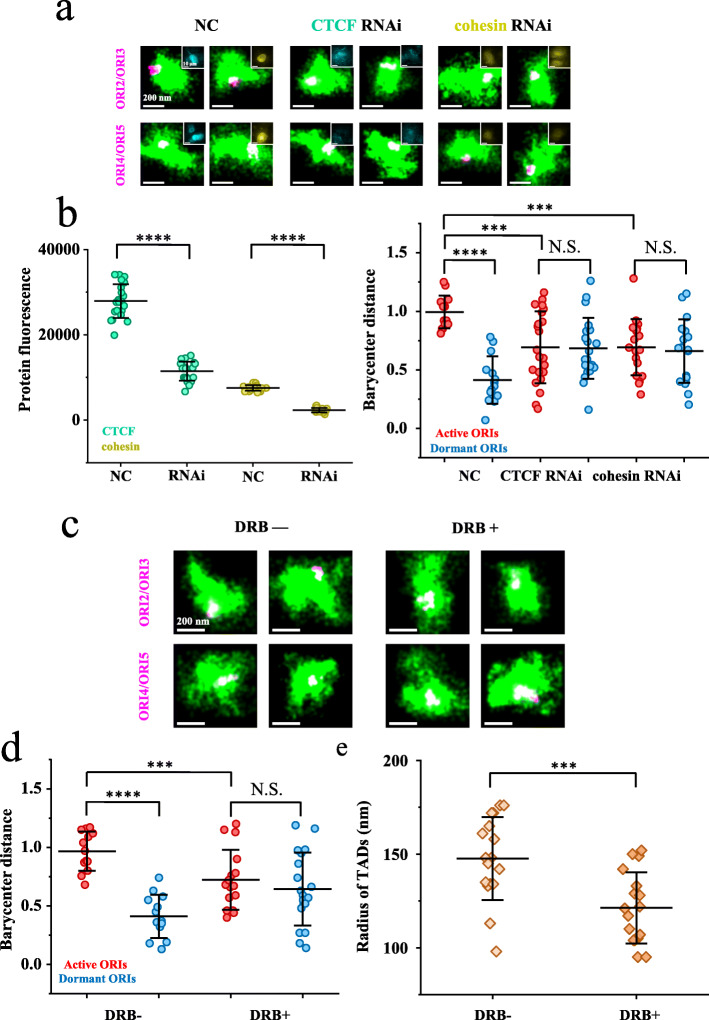
The spatial distribution of replication origins within the TADs at the G1/S transition is dependent on CTCF, cohesin, and transcription. **a** Representative STORM images of origins (purple) in TAD1 (green) after treatment of cells with the indicated siRNAs. Conventional images indicate the concentration of CTCF (cyan) or cohesin (yellow) in the nucleus. **b** Left panel, efficiency of RNAi indicated by fluorescence of CTCF or cohesion. Right panel, barycenter distances between high-efficiency or low-efficiency origins in TAD1 after treatment of cells with the indicated siRNAs. Portions of the two signals that overlap are shown in white. **c** Representative STORM images of origins (purple) in TAD1 (green). Left: no DRB. Right: with DRB. **d** Barycenter distances between high-efficiency/low-efficiency origins and TAD1 with or without DRB treatment. **e** Radius of TAD1 treated with or without DRB. For lines and statistics in **b**, **d**, and **e**, see the description in the legend of Fig. 1 (n ≥ 10 cells). 3D results are shown in Fig. S13

Next, we explored the factors that are responsible for differential origin localization in the TAD. We first examined the effects of CTCF and cohesin. Upon down-regulation of CTCF or cohesin using RNAi (Fig. 3a, insets and Fig. 3b, left panel), we found that the high-efficiency origins (ORI2 and ORI3) were not relocated to the TAD periphery at the G1/S transition in both 2D and 3D images (Fig. 3a and Additional file 1: Figure S13a). More importantly, the barycenter distances of either high-efficiency origins or low-efficiency origins relative to TAD1 became similar with that of randomly distributed foci (about 0.7) (Fig. 3b and Additional file 1: Figure S13b). Such effect was likely due to the scrambling of chromatin structure within the TADs upon loss of CTCF or cohesin, which is in line with the Hi-C data that depletion of either cohesin or CTCF eliminates loops [53, 54]. These results suggested that the relocation of replication origins in the G1 phase is dependent on chromatin looping mediated by CTCF and cohesin in the TAD.

Previous studies have shown that in the G1 phase, transcription activity is generally high in early RDs and high-efficiency origins abut actively transcribed genes [40, 55]. Transcription has also been found to fundamentally influence chromatin structures at different levels and through various mechanisms, including nucleosome disassembly, enhancer-promoter loop formation, transcript cis-interaction, CTCF and cohesin displacement, gene relocation, and transcription factory formation [56–58]. Moreover, in a recent study, Gilbert and his colleagues identified cis-acting elements, namely early replicating control elements (ERCEs), which regulate the replication timing and the structure of TADs [59]. Importantly, ERCEs have properties of enhancers or promoters, implicating a fundamental role of transcription in orchestrating genome replication and chromatin architecture. Therefore, given that the origin relocation takes place in the G1 phase, we next examined whether transcription in the G1 phase contributes to chromatin structural re-organization and origin relocation within the TADs.

To do so, we treated cells with transcription elongation inhibitor 5,6-dichloro-1-β-d-ribofuranosyl-benzimidazole (DRB) [60] from the mid-G1 phase to the G1/S transition, after which we labeled TAD1 and its replication origins using Oligopaint probes. Interestingly, upon transcription inhibition by DRB treatment, high-efficiency origins ORI2 and ORI3 were no longer found to relocate to the periphery of the TAD at the G1/S transition in both 2D and 3D images (Fig. 3c and Additional file 1: Figure S13c) and had barycenter distances similar to those of low-efficiency origins (Fig. 3d and Additional file 1: Figure S13d). Moreover, the radius of TAD1 at the G1/S transition in DRB-treated cells was found to be smaller than that in normal cells (Fig. 3e and Additional file 1: Figure S13e) and similar with that observed in the G1 phase (Fig. 2d and Additional file 1: Figure S11c). This observation suggested that transcription de-compacts the chromatin structure of TADs. As DRB inhibits transcription globally and may repress the expression of genes correlated with DNA replication or chromatin structure organization, we then tried to modulate the expression of particular genes within TAD1. Specifically, we applied CRISPRi [61], a CRISPR technique that can block transcription of targeted genes, to repress the transcription of *ATP13a2*, an actively transcribed gene in TAD1. Indeed, we observed that in the *ATP13a2* repressed cells, both the barycenter distance of high-efficiency origins and the radius of TAD1 became smaller (Additional file 1: Figure S14). Taken together, these results suggested that transcription-dependent chromatin structural re-organization within the TADs exposes a subset of origins to the physical boundary of a TAD, which are preferentially used for replication initiation.

### Replication elongation factor PCNA surrounds TADs both in the G1 and G1/S phases

**Fig. 4:**
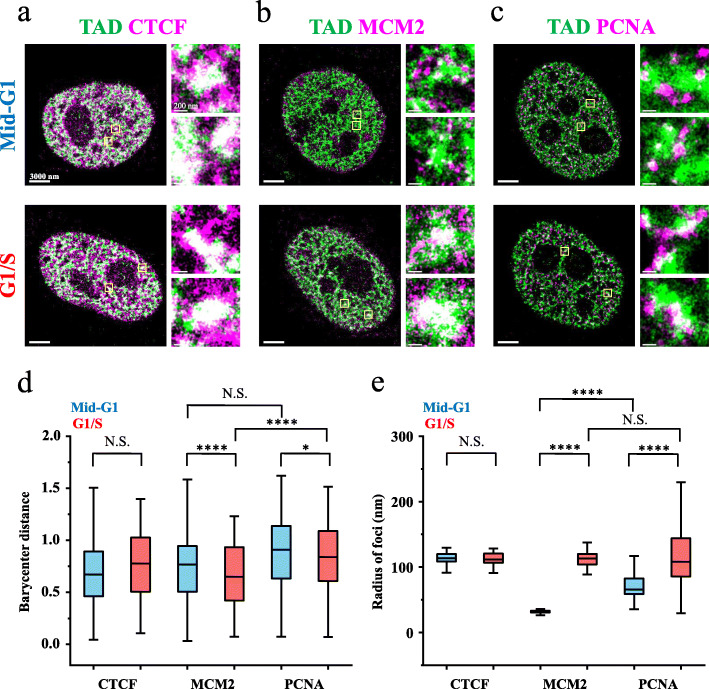
Spatial distributions of CTCF, MCM2, and PCNA relative to the early replicating TADs in the G1 and G1/S phases. **a**–**c** Representative STORM images of CTCF, MCM2, and PCNA labeled by immunostaining (purple) and metabolically labeled TADs (green). Cells were fixed and labeled in the mid-G1 phase (upper) or G1/S phase (lower). The areas inside the yellow squares are shown at higher magnification next to each nucleus. Portions of the two signals that overlap are shown in white. **d** Barycenter distances between CTCF, MCM2, or PCNA with the TADs in the mid-G1 phase or G1/S phase. **e** Radii of CTCF, MCM2, or PCNA foci in the mid-G1 phase or G1/S phase. For lines and statistics in **d** and **e**, see the description in the legend of Fig. 1 (n = 10 cells)

To answer why origins located on the physical boundary of a TAD get preferentially used for DNA replication, we examined the spatial distribution of replication machinery relative to individual TADs at the G1 phase and G1/S phases by imaging proliferating cell nuclear antigen (PCNA) [62]. As a control, we also monitored the distribution of minichromosome maintenance complex component 2 (MCM2) and CTCF, respectively. Provided that metabolically labeled RDs merge well with FISH-labeled TADs in the early S phase (Fig. 1 and Additional file 1: Figure S5/S8), to label early replicating TADs and protein factors in the same cell, TADs were first labeled by supplying cells with EdU for 45 min immediately after the release of replication arrest at the beginning of the S phase; in the next cell cycle, the cells were fixed and immunostained at either the mid-G1 or G1/S phase. The EdU-labeled TADs became larger from the mid-G1 phase to the G1/S transition (Additional file 1: Figure S15), in line with the observation of FISH-labeled TAD1 (Fig. 2d). As a scaffold factor of TADs, CTCF formed large foci (Fig. 4a) and neither their spatial distribution relative to the TADs (Fig. 4d) nor their sizes (Fig. 4e) were found to change in the G1 phase. Interestingly, despite the constant sizes of the CTCF foci, both the single-molecule detection counts (Additional file 1: Figure S16a) and the molecule density (Additional file 1: Figure S16b) in the CTCF foci were reduced from the mid-G1 phase to the G1/S transition, suggesting that CTCF dissociated from DNA during the transcription-dependent chromatin structural re-organization process. This STORM-based finding is consistent with a previous single-molecule study showing that binding of CTCF to chromatin decreases from the G1 phase to the S phase [63], as well as a Hi-C study showing that transcription elongation can disrupt CTCF-anchored chromatin loops [58].

In contrast to CTCF, MCM2 and PCNA showed drastically different patterns. In the G1 phase, MCM2 formed small clusters that distributed relatively around the TADs (Fig. 4b, d). Intriguingly, at the G1/S transition, MCM2 foci became significantly larger (Fig. 4b, e) and distributed more toward the center of the TADs (Fig. 4b, d). Quantitative analyses of the foci showed that, while the single-molecule detection counts in the MCM2 foci increased from the mid-G1 phase to the G1/S transition (Additional file 1: Figure S16a), the molecule density decreased (Additional file 1: Figure S16b). This observation suggested that MCM2 gradually became associated with chromatin in the G1 phase, in line with the results by Gilbert and his colleagues [38]. As a replication elongation factor of the initiation complex, PCNA binds a subset of origins with the pre-IC complex and recruits DNA polymerases. In the G1 phase, we found that PCNA formed small clusters around the TADs (Fig. 4c, d). However, unlike MCM2, the PCNA foci remained surrounding the TADs at the beginning of the S phase (Fig. 4c, d). This spatial distribution of PCNA clusters provides a possible explanation for preferential initiation of origins at the TAD periphery. Moreover, from the mid-G1 phase to the G1/S transition, the size of the PCNA foci was nearly doubled (Fig. 4c, e) with both the single-molecule detection counts and the molecule density in the foci increased dramatically (Additional file 1: Figure S16a/b). These data suggested that PCNA was gradually recruited to chromatin DNA, consistent with the previous reports that PCNA clusters are much more visible by live cell imaging in the S phase in comparison with the G1 phase [64–67].

## Discussion

Here, we unveiled a new mechanism for replication origin selection by directly visualizing individual TADs and the spatial distribution and dynamics of replication origins in the TADs using super-resolution imaging. We first found that replication initiation generally takes place separately at the spatial boundary of the TAD (Fig. 1 and Additional file 1: Figure S5). Next, we discovered that origins undergo relocalization along with the structural re-organization within the TAD in the G1 phase, and the origins that either relocate to (e.g. ORI2 and ORI3) or remain at (e.g. ORI1) the spatial boundary of the TAD are of higher replication efficiency (Fig. 2 and Additional file 1: Figure S11). Importantly, we found that chromatin structural re-organization within the TADs is driven by disruption of chromatin loops during transcription elongation [58] in the G1 phase (Fig. 3 and Additional file 1: Figure S13). Lastly, we observed that the major replication machinery protein PCNA, which was previously found to be immobile in the S phase [64–67], remains surrounding the TADs from the mid-G1 phase to the S phase and provides the origins exposed at the spatial boundary of a TAD with a better chance of accessing the replication machinery.

### The “Chromatin Re-organization Induced Selective Initiation” (CRISI) model

**Fig. 5:**
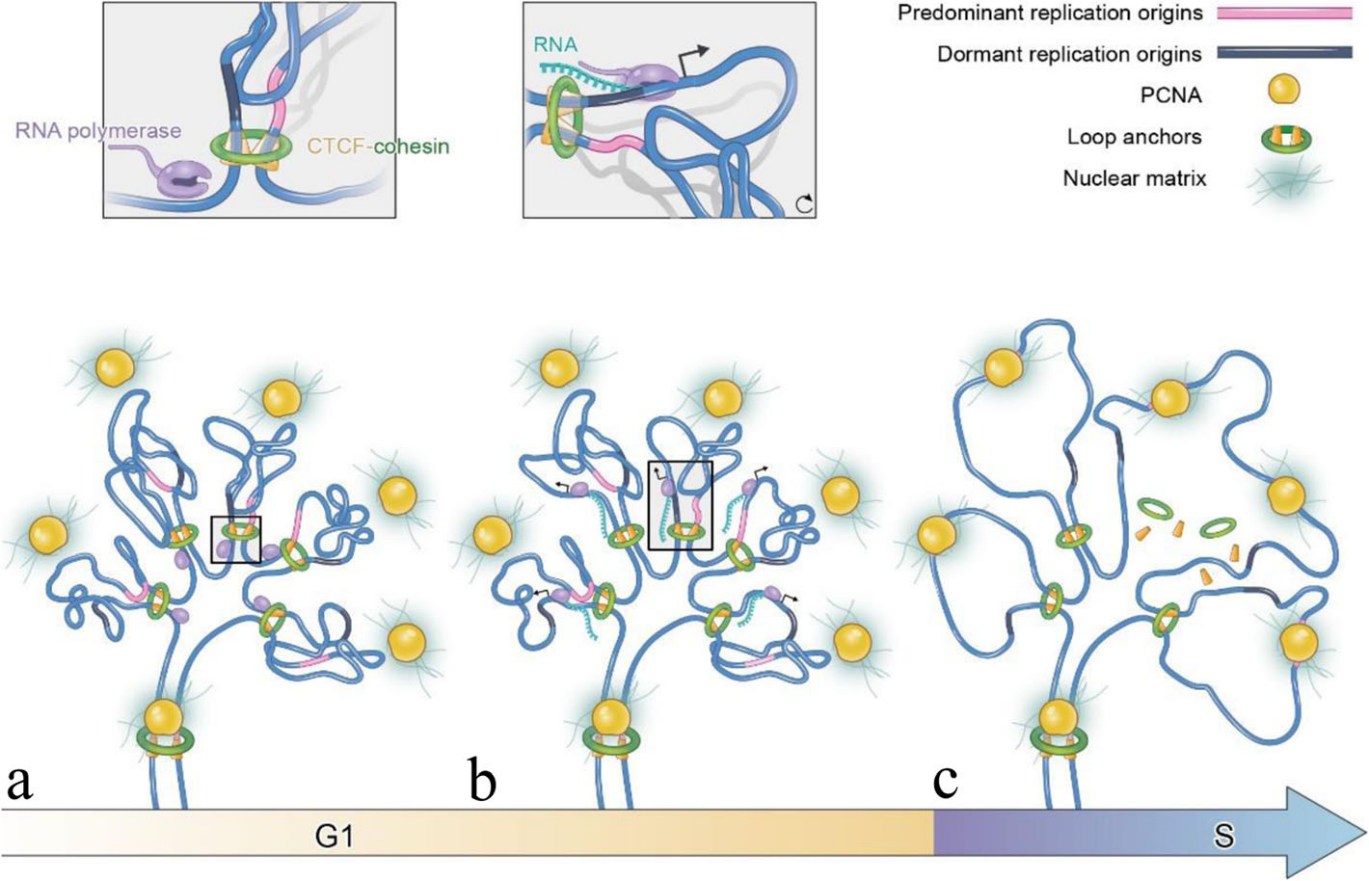
“Chromatin Re-organization Induced Selective Initiation” (CRISI) model for selective initiation of DNA replication origins. **a** In the early G1 phase, the spatial distributions of potential replication origins (gray ribbons) are relatively even in the TAD. The TAD comprises several chromatin loops (blue) organized by CTCF and cohesin at the loop anchors (green rings). PCNA clusters (yellow balls) surrounding the TAD are bound to the nuclear matrix (hazed light blue straws). **b**, **c** With transcription proceeding, the chromatin loops undergo structural re-organization along with chromatin domain de-compaction in the G1 phase, exposing a subset of the origins to the periphery of the TAD (pink ribbons). Note that the origin at the sequence boundary of the TAD remains at the TAD periphery in the G1 phase. These peripheral origins are more accessible to the surrounding PCNA clusters and thus become high-efficiency origins for the initiation of DNA replication at the periphery of the TAD. The areas inside the black squares in **a** and **b** are shown at higher magnification above

Based on our results, we propose a “Chromatin Re-organization Induced Selective Initiation” (CRISI) model (Fig. 5) for replication origin selection. The CRISI model suggests that the spatial localization of an origin in a TAD determines its replication efficiency. Dynamically, in the early-to-mid G1 phase, all potential origins distribute homogeneously in the TAD (Fig. 5a). Upon the onset of transcription, the chromatin loops in the TAD are de-compacted and some loop anchors are disrupted, leading to a subset of origins relocalizing from the inside of the TAD to the periphery (Fig. 5b, c). Meanwhile, PCNA forms clusters that remain around the TADs from the mid-G1 phase to the G1/S transition. The peripherally and separately located origins are more accessible to the surrounding PCNA clusters and thus become high-efficiency origins (Fig. 5c). The distribution of high-efficiency origins in TADs in our CRISI model is in contrary with the classic Rosette model, which proposes that the high-efficiency origins cluster and co-fire in the chromatin domain [4].

Recently, based on the observation that nearly all replicons are spatially separated at the beginning of the S phase, Cardoso and his colleagues also questioned the Rosette model [21]. They proposed a stochastic, proximity-induced replication initiation model, describing induced domino-like origin activation that may lead to the temporal grouping of high-efficiency replicons within a chromatin fiber [26]. Nevertheless, as the replication origins were not imaged along with the chromatin domains, how the origins are spatially organized in the chromatin domain and whether the distribution can differentiate the origin efficiency were not known. In the current work, we realized the first direct visualization and quantification of the relative localization and organization of replication origins within individual TADs. Given that a TAD is typically ~ 200 nm in radius (Fig. 2d and Additional file 1: Figure S11c) and the difference in barycenter distances between high-efficiency and low-efficiency origins is less than 100 nm (Fig. 2c and Additional file 1: Figure S11b), such quantification would require simultaneous imaging of both individual TADs and the associated replication origins with nanometer spatial resolution. Therefore, 3D STORM with 20 nm lateral and 50 nm axial resolution would be more suitable for such analyses.

Regarding the constrained mobility of PCNA clusters in the nucleus, we speculate three possible mechanisms that are not mutually exclusive. Firstly, PCNA and the replisomes are giant complexes binding DNA with low diffusive mobility. Secondly, PCNA and the replisomes may be attached to the nuclear matrix (Fig. 5c), which is supported by an immunoelectron microscopy study showing that DNA polymerase ɑ, PCNA, and nascent DNA are colocalized in nucleoskeleton bodies [68]. Thirdly, the proteins comprising replisome complexes might form liquid condensates that phase-separate from TADs [69]. These possibilities would be interesting subjects for future studies.

That the chromatin structural dynamics within the TADs make origins accessible to immobile PCNA clusters provides an interesting viewpoint to understand protein-DNA interactions in the nucleus, which are commonly considered to be based on diffusive search of proteins such as transcription factors on chromatin DNA [70, 71]. Such mechanisms may be involved in various nuclear functions. For example, during DNA damage repair, ATM is restricted at the double-strand breaking (DSB) site while phosphorylation of H2AX by ATM spreads over a domain [72]. The discrepancy between the distribution of the kinase (ATM) and its product (γH2AX) can be explained by the local movements of the chromatin fiber inside the TAD which bring distant nucleosomes to spatial proximity of ATM [72]. In the future, other imaging methods such as the sequential imaging approach (Hi-M) may be combined with oligoSTORM to further investigate chromosome organization and functions in single nuclei [73].

### Replication, transcription, and chromatin structure

It has been known for many years that transcription is profoundly related to replication [74, 75]. However, while transcription is known to be highly correlated with the replication timing of TADs [59, 76], it is unclear whether transcription regulates origin selection within individual TADs. Intriguingly, although the genetic and epigenetic signatures of high-efficiency origins mapped by various methods seem quite different and hierarchical [2], they are mostly markers of active transcription and interdependent in the context of transcription. Transcription has been reported to change chromatin structure at different levels. Our imaging data reveal that the transcription activities can displace CTCF from the TADs (Additional file 1: Figure S16) and de-compact the chromatin domain (Figs. 2d and 3e), consistent with the previously reported Hi-C data [50, 58]. These effects, together with other transcription-induced changes in nucleosomes, chromatin fibers, and enhancer-promoter loops, re-organize the chromatin structures within the TADs to relocate a subset of origins to the TAD periphery. Consequently, these origins possess higher replication efficiency for being more accessible to the peri-TAD PCNA clusters. While our results explain why a subset of origins is preferentially activated in a TAD, it should be noted that how specific origins are relocated to the TAD periphery by transcription activity is unclear and requires further investigation. The transcription-dependent CRISI model predicts that enhancing transcription activity should increase the selectivity of replication origins, while repressing transcription should cause the opposite effect. Indeed, two recent studies of replication initiation in particular genes showed that enhanced transcription leads to more selective initiation of origins [55, 77] while transcription inhibition causes more origins to be used for replication [78, 79].

Encountering between the transcription and replication machineries is a major intrinsic source of genome instability [74, 80]. Therefore, how cells prevent or resolve the transcript-replication conflicts has been an important question. One major mechanism is to temporally separate transcription and replication for the same genomic regions [81]. Our data suggested that the replication machineries are confined around the TAD and spatially separated from the transcription machineries, which mainly function within the TAD. Therefore, our work provides a new mechanism for cells to avoid the conflicts between replication and transcription based on spatial/topological separation.

In summary, the CRISI model demonstrates important coordination among DNA replication, transcription, and chromatin structure, which reconciles the discrepancy of different signatures for origin efficiency. Lastly, our work also provides new insights into how 3D genome structural dynamics, particularly the intra-TAD physical structures, may regulate other nuclear processes on chromatin templates such as DNA repair, adding a new layer of understanding to chromatin structure and functions.

## Methods

### Cell culture and manipulations

The HeLa-S3 cell line (PubMed ID: 5733811) and the human retinal pigment epithelium (HRPE) cell line were obtained from Dr. Wei Guo, University of Pennsylvania. The U2OS cell line and the MDA-MB 231 cell line were purchased from the Cell Bank of Chinese Academy of Sciences. Cell lines were authenticated using Short Tandem Repeat profiling using ANSI/ATCC ASN-0002-2011 guidelines and tested Mycoplasma negative according to the MycAwayTM-Color One-Step Mycoplasma Detection Kit (Yeasen). HeLa S3 cells were grown in 10-mm glass-bottom imaging dishes (Cellvis #D35-10-1-N) with 2-mL modified medium (high-glucose DMEM, Thermo Fisher Gibco #10569-044) supplemented with 10% (v/v) fetal bovine serum (Thermo Fisher Gibco #10091-148) and 100 U/mL penicillin-streptomycin antibiotics (Thermo Fisher Gibco #15070-063) under regular cell culture conditions (37 °C, 5% CO2, humidified atmosphere). Cells were passaged with the proportion of 1:8–1:10 by trypsin (Life Technologies #25200-056) every 3 days or when they reached 80% confluence.

HeLa cells were synchronized to the G1/S boundary by two rounds of blocking as described previously [7, 38]. In the first blocking period, HeLa cells at approximately 25% confluence were treated with 2 mM thymidine (sigma #T1895-5G) for 15 h and then released to a fresh medium for 10 h. In the second blocking period, cells were arrested by 2 μg/mL aphidicolin (sigma #A0781-1MG) for 15 h.

For FISH labeling of the TADs and metabolic labeling of replicating DNA in Fig. 1a, synchronized cells were labeled by EdU for the designated durations upon release into the S phase, followed by Click reactions and FISH labeling. For double metabolic labeling of replicating DNA in Fig. 1c, synchronized cells were labeled by BrdU for 45 min and then transferred to a fresh medium. Cells were then synchronized by aphidicolin, labeled with EdU for the designated durations upon release into the next S phase, and fixed by 4% paraformaldehyde (PFA). For double FISH labeling of the TADs and origins at the G1/S transition in Fig. 2b, synchronized cells were released with fresh medium and synchronized again until they were fixed at the next G1/S phase for FISH labeling. For double FISH labeling of the TADs and origins at the mid-G1 phase in Fig. 2b, cells were synchronized in mitosis as previously described [51]. In brief, after release from the aphidicolin block, cells were transferred to a fresh medium and nocodazole was added to a final concentration of 0.1 μg/mL. After incubation for 10 h, mitotic cells were collected by mechanical shaking and transferred to a fresh medium. After 7 h, cells were fixed at 5 h in the G1 phase, i.e., mid-G1 phase, for FISH labeling. For Fig. 3a, after down-regulation of proteins, cells were synchronized and fixed at the G1/S transition for double FISH labeling. For Fig. 3c, synchronized cells were released, after which DRB was added at a final concentration of 100 μM from 5 h in the G1 phase to the S phase in the third cell cycle. Cells were fixed at the G1/S transition for double FISH labeling. For metabolic labeling of early replicating TADs and immunolabeling of proteins at the beginning of the S phase in Fig. 4, synchronized cells were labeled by EdU for 45 min and then transferred to a fresh medium. Cells were synchronized again until they were fixed at the next G1/S phase for protein immunolabeling. For metabolic labeling of TADs and immunolabeling of proteins at the mid-G1 phase in Fig. 4, synchronized cells were labeled by EdU for 45 min and then synchronized in mitosis as previously described [51]. In brief, after release from the aphidicolin block, cells were transferred to a fresh medium and nocodazole was added to a final concentration of 0.1 μg/mL. After incubation for 10 h, mitotic cells were collected by mechanical shaking and transferred to a fresh medium. After 7 h, cells were fixed at 5 h in the G1 phase, i.e., mid-G1 phase, for protein immunolabeling.

For the Extended Data Figures, cells were manipulated using the same methods used for the experiments shown in the related figures. The procedures used for FISH and immunolabeling are described in detail below.

### Metabolic replication labeling

At specific times after releasing the cells from the G1/S boundary, replication foci were labeled by directly adding thymidine analogs EdU (Click-iT® Plus Alexa Fluor® 647 Picolyl Azide Toolkit, Thermo Fisher Invitrogen #C10643) or BrdU (Abcam #ab142567) for a designated time period. The final concentration of EdU and BrdU was 10 μM. dUTP-atto-550 (Jena Bioscience #NU-803-550-S) was delivered into the nucleus using the FuGENE 6 transfection reagent (Promega #E2691) according to the manufacturer’s instructions. For non-synchronized cells, BrdU labeling was conducted for 1 h followed by 1 h EdU labeling. BrdU was further labeled by immunostaining as described in detail below. Alexa Flour 647 was conjugated by click reaction according to the manufacturer’s instructions. In brief, after fixation and washing, samples were treated with 1% Triton in 5% BSA for 30 min. The samples were washed three times, after which a fresh reaction cocktail was added for 30 min. The reaction cocktail was made by sequentially mixing 1× Click-iT reaction buffer, copper protectant, Alexa Fluor picolyl azide, and reaction additive buffer. Finally, the samples were washed three times.

### Sequencing data

The replication timing profile remapped to hg38 was obtained from the Replication Domain Genome Browser of the Gilbert lab (https://www2.replicationdomain.com/ genome_browser). Hi-C data were obtained from ENCSR693GXU and further analyzed using previously described methods [82]. In brief, Hi-C data was analyzed using HiCUP (v0.5.8) using default parameters, then duplicates were removed using samtools (v1.0.0). Analyzing Hi-C from the Homer suite (v4.8) was used for the coverage (-window 1000000) and DLR (distal-to-local ratio) calculation (-dlrDistance 3000000). For BrdU-seq, in brief, cells were synchronized to the beginning of the S phase and released, after which BrdU was supplied for 10 min at 0 min, 1 h, 3 h, and 6 h into the S phase. Sample preparations and data analyses for the ChIP-seq experiments were performed following standard protocols [48]. ChIP-seq data sets of CTCF and cohesions (SMC1 and SMC3) were initially analyzed as described for histone ChIP-seq data analysis. Summits of the CTCF peaks overlapped with cohesion peaks were remained for the distribution analysis of ORC1 peaks. Sequencing data was remapped to hg38.

### Replication domain labeling by fluorescence in situ hybridization

#### Probe design

To label target genomic regions, corresponding oligonucleotide probes were designed using Oligominer (https://github.com/brianbeliveau/Oligominer) following online instructions and methods described in a previous work [27]. All templates for primary hybridization probes synthesis were 102 nt in length and contained the following components: (i) one 32-nt central sequence targeting the genomic region of the target, (ii) one 30-nt flanking sequence to hybridize with the secondary probes, and (iii) two 20-nt flanking primer binding sequences to amplify the probes through polymerase chain reaction (PCR). For probe synthesis, a 20-nt T7 promoter sequence was added to the 5′ end of each forward primer for in vitro transcription. The primers used for probe synthesis and the secondary probe sequences are listed in Additional file 4: Table S3.

#### Probe synthesis

The probes were amplified from a template oligopool synthesized by Hongxun Biotech (Suzhou, China). All primers were synthesized by Invitrogen. The probe synthesis was performed by a five-step enzymatic amplification procedure as follows:

Step 1: Through 26 cycles PCR with the above primers, 2 μL of the template oligopool was amplified in a 50-μL reaction mix (Phanta Max Super-Fidelity DNA Polymerase, #P505-d2). The final PCR product was column-purified (Zymo DNA Clean and Concentrator, DCC-5).

Step 2: To obtain the target products at a high concentration, the purified DNA products were collected and used as the template to repeat step 1. Next, the purified PCR products were diluted to a concentration of 100 ng/μL.

Step 3: The diluted DNA was converted into RNA via in vitro transcription (HiScribe™ T7 High Yield RNA Synthesis Kit NEB, #E2040S). Each 33-μL reaction contained 17 μL of template DNA obtained as described above, 2.5 μL (10 mM) of each NTP, 3 μL of 10× reaction buffer, 1 μL of RNase inhibitor (Promega RNasin® Plus RNase Inhibitor #N2615), and 2 μL T7 polymerase. The reaction was incubated at 37 °C for 5 to 6 h.

Step 4: RNA products from the in vitro transcription reaction were converted back into single-stranded DNA via reverse transcription (MAN0012047 TS Maxima H Minus Reverse Transcriptase, Thermo Fisher #EP0751). Each 70-μL reaction contained 33 μL of the RNA products obtained in step 3, 10 μL dNTPs, 14 μL RT buffer, 1 μL RT enzyme, 1 μL RNase inhibitor (Promega RNasin® Plus RNase Inhibitor #N2615), 1 μL ddH2O, and 10 μL 40 μM TAMRA-labeled reverse transcription primer. The reaction was incubated at 50 °C for 1.5 h.

Step 5: The template RNA obtained in step 4 was removed by incubating it with 50 μL of 0.25 M EDTA and 0.5 M NaOH at 95 °C for 10 min. Next, the products were put on ice and immediately purified by column purification (Zymo Research, #D4006). The resulting single-strand DNA probes were eluted twice in 20 μL of ultra-pure water. Probes purified in this manner can be stored temporarily at −20 °C or at −80 °C for several months. The quality of the probes was determined by measuring the sample concentration (200–300 ng/μL) and the fluorescence absorbance of tagged dyes.

#### In situ hybridization

Step 1: Sample preparation

Cultured cells were prepared for fluorescence in situ hybridization (FISH) as described in previous works [27, 36]. Briefly, cells were fixed on dishes with 4% PFA, washed three times in PBS, and incubated in 1 mg/mL NaBH_4_ solution for 7 min to quench background signals. If FISH is combined with other labeling methods, such as immunostaining or click reaction, FISH should be performed after other labeling steps, and the quenching step must be skipped. Cells were then permeabilized with PBS containing 1% Triton X-100 (PBST) for 10 min and washed twice in the same buffer. To remove all RNA, cells were treated with 100 μg/mL RNaseA in 1× PBST for 45 min at 37 °C, after which they were washed three times in PBST. The samples were incubated with 0.1 M HCl in 1× PBST for 10 min, followed by two washes in 2× SSCT. To unfold chromatin, 50% formamide in 2× SSCT was added to the samples for at least 4 h or overnight.

Step 2: Fluorescence in situ hybridization

The cells were incubated with 50% formamide in 2× SSCT at 78 °C for 10 min and then put on ice immediately. Next, the samples were dehydrated in 70%, 85%, and 100% icy ethanol successively for 1 min each. Meanwhile, 5 μL of the synthesized primary probes and 1 μL of the synthesized secondary probes (100 μM) were mixed with 35 μL of 100% formamide at 37 °C in a 1500 rpm vortex for 15 min. At the same time, 20% Dextron/4× SSC was shaken at 37 °C in a 1500 rpm vortex for 15 min. Next, 41 μL of formamide with probes and 35 μL of 20% Dextron/4× SSC added by 1 μL of triton were mixed and shaken for 30 min at 37 °C in a 1500 rpm vortex. The fluorescent probes were kept in darkness. The 77-μL hybridization cocktail was incubated at 86 °C for 3 min and immediately put on ice before the hybridization. The processed sample was incubated with the hybridization cocktail at 86 °C for 3 min, followed by hybridization in a wet box at 37 °C for 16–20 h.

Step 3: Post-hybridization washes

The samples were briefly washed three times with 2× SSCT. Next, the samples were washed three times with 2× SSCT at 60 °C for 10 min. The signal and background noise of the samples were checked by microscopic imaging. If the background noise was high, the samples were washed several times with 2× SSCT and 50% formamide at 37 °C for 1 min each time to obtain an appropriate signal-to-noise ratio. Finally, the sample was stored in 2× SSCT prior to STORM imaging. All reagents mentioned in this section of the methods were purchased from Sigma.

### Immunostaining of BrdU, PCNA, MCM, and CTCF

Cells were fixed by 4% PFA for 15 min and then blocked and permeabilized in 5% bovine serum albumin (BSA) containing 1% Triton X-100 (Sigma-Aldrich) for 30 min, after which the cells were washed three times in PBS containing 1% (v/v) Triton. All primary antibodies (CTCF, Abcam #ab128873; PCNA, CST #13110 T; MCM, CST #3619 T; BrdU, Abcam #ab152095) were diluted (1:200) in 5% BSA containing 1% Triton. Next, cells were incubated with primary antibodies for 1 h in darkness, followed by three washes in PBS (5 min, 10 min, and 5 min). Cells were incubated with 1:50 dye-conjugated secondary antibody for 1 h followed by four washes in PBS (5 s, 5 min, 10 min, and 5 min). After the cells were post-fixed in 4% PFA for 10 min, they were washed three times with PBS (15 s each) and stored at 4 °C. All of the steps described above were performed at room temperature.

### RNA interference

siRNA oligos were transfected into cells with Lipofectamine 2000 (Invitrogen #11668-027) at a final concentration of 100 nM according to the manufacturer’s instructions. As previously reported [25], two rounds of transfection were separated by 24 h. The first round of interference was conducted when the cells reached approximately 30% confluence. Cells were released to a fresh medium after the transfection, which lasted for 5 h. After approximately 20 h, RNA interference was conducted again when the cells reached approximately 50% confluence. After the cells were released to a fresh medium for approximately 4 h, thymidine was added and two rounds of synchronization were performed as described above. Control transfections were performed with scrambled control siRNA. siRNAs targeted to CTCF and Rad21 were designed as follows:

CTCF: GGAGCCUGCCGUAGAAAUUTT (sense)

AAUUUCUACGGCAGGUCCTC (anti-sense)

Rad21-1: GGUGAAAAUGGCAUUACGGTT (sense)

CCGUAAUGCCAUUUUCACCTT (anti-sense)

Rad21-3: GACAUGUUAGUAAGCACUACUACUU (sense)

AAGUAGUAGUGCUUACUAACAUGUC (anti-sense)

Rad21-1 and Rad21-3 were combined in equal proportions.

Control: CGUACGCGGAAUCUUCGATT (sense)

UCGAAGUAUUCCGCGUACGTT (anti-sense)

The efficiency of siRNA knockdown in individual cells was checked by immunostaining.

### STORM imaging

STORM imaging was performed on a custom-built inverted microscope (IX83, Olympus) using wide-field excitation mode as previously reported [31]. The microscope utilized a 100×, 1.40 NA oil-immersion objective lens (UPlanSApo 100×, 1.40 NA, Olympus). A multiband dichroic filter (Chroma) was used to reflect the lasers while ensuring transparency to the fluorescence of the sample. Extra emission filters (Chroma) were added to separate the fluorescence in each channel, respectively. Images were recorded with an EMCCD (Ixon+ EMCCD, Andor) at 256 × 256 pixels (160 nm per pixel).

An extra NIR laser was used to achieve perfect focusing. This weak laser beam was separated by a 50:50 prism, after which two mirrors reflected the resulting beams back to the prism for merging into the same direction. After these beams were collimated to the objective from opposite edges, they were reflected back and focused onto a NIR camera, resulting in two separated points. The objective was mounted on a nanopositioning system (Nano F100S, Mad City Labs), with which the distance change from two infrared points leaded by the sample drift that could be feed-back controlled.

For 3D imaging, a cylindrical lens was inserted between the microscope and the camera to achieve optical aberration. Before imaging, a calibration curve was generated by imaging individual dye molecules while scanning the sample in the *z* direction.

Silicon dioxide beads (3 μm diameter) were incubated with the sample for 0.5–2 h (1:500 ratio), followed by three washes with PBS to remove unstable beads. Next, the PBS was replaced with imaging buffer (10% (w/v) glucose, 20 mM NaCl, 100 mM Tris/HCl, 600 μg/mL glucose oxidase, 60 μg/mL catalase, 1% (v/v) beta-mercaptoethanol). The dish was placed on the objective stage after the microscope was configured. With the appropriate focal plane, the targeted cells were chosen based on three features: qualified infrared focal locking signal, clear images in the 561-nm and 647-nm channels, and at least one stable bead along with the cell in a bright field.

Fluorophores were activated by a 405-nm laser (OBIS, Coherent) and excited by a 561-nm laser and 647-nm laser (MBP laser), respectively. At first, cells were chosen and conventional images were collected with a 647-nm or 561-nm solid-state laser. Next, the immobile silicon dioxide beads were imaged in a bright field for the drift correction process. The probes were turned rapidly to the dark state by a 561-nm or 647-nm solid-state laser, after which they were imaged with 561-nm or 647-nm laser excitation (1000 mW, > 3 kW/cm^2^) and weak 405-nm laser activation (< 2 W/cm^2^, increased from zero manually, maintaining a roughly constant density of activated molecules). STORM imaging movies were obtained with 100-Hz imaging speed and consisted of 60,000 frames in total for the 647-nm and 561-nm channels, respectively. Interlaced bright field images of the 561-nm and 647-nm channels taken as described above were utilized for drift correction between these two channels.

After imaging, STORM imaging buffer was removed by three washes in PBS, after which the samples were stored in PBS at 4 °C.

### STORM image analysis

For single-molecule localization, fluorophores were fitted and localized by Insight3 (gift from Prof. Bo Huang, UCSF) using the Gaussian fit method. Comparison of the *x* and *y* widths of a single detection with a reference calibration curve as mentioned above was used to determine the *z*-position. Drift correction and data rendering were performed using custom code (MATLAB 2016a). Bright field images of SiO_2_ beads were used for cross-correlation to obtain the drift correction curve [23]. 2D and 3D STORM images were further clustered by SR-Tesseler and DBSCAN, respectively. Data involving non-synchronized cells and multiple cell lines were analyzed using ThunderSTORM [83] and custom Python codes.

We utilized the pointillist nature of STORM data for calculation. Thus, each localization for calculation was treated as a point with “mass” of 1 unit. In this way, the barycenter of a TAD or a replication site was calculated as the mass center of all single-molecule localizations it contains.

For the SR-Tesseler analysis, software was downloaded from the authors’ website http://www.iins.u-bordeaux.fr/team-sibarita-SR-Tesseler. A Voronoi diagram was created, after which clusters were identified by the proper density factors. Different density factors were evaluated for the identification of TADs and small foci such as protein clusters or replication origins (Additional file 1: Figure S4). For analyzing FISH or metabolically labeled TADs, the density factor was set to 3. For analyzing FISH or metabolically labeled replication origins or initiation sites, the density factor was set to 20. For analyzing protein clusters, the density factor was set to 3.5. The following information was directly exported: detection, density, major axis, diameter, and barycenter of x and y. We defined the barycenter distance to describe the spatial distribution of replication foci, replication origins, or proteins in TADs. Taking FISH-labeled replication origins and TADs as an example, the barycenter distance is the spatial barycenter distance between the origins and the TAD divided by the radius of the TAD. To ensure that the origins belonged to the TAD, an origin was analyzed only if the spatial barycenter distance between the origin and its nearest TAD was shorter than half of the length of the major axis of the TAD.

To assess our experimental results, we calculated the expected barycenter distance for randomly distributed foci in a polar coordinate representation:

$$ \overline{r}=\frac{\underset{0}{\overset{2\pi }{\int }} d\varphi \underset{0}{\overset{\pi }{\int }} d\theta \underset{0}{\overset{R}{\int }} d r\ {r}^3\ {\cos}^2\theta }{\underset{0}{\overset{2\pi }{\int }} d\varphi \underset{0}{\overset{\pi }{\int }} d\theta \underset{0}{\overset{R}{\int }} d r\ {r}^2\ \cos \theta }=\frac{3\pi }{16}R $$

where $\overline{r}$ denotes the average spatial distance between small foci within the TAD and *R* represents the sampling radius, which, in our case, equals half the length of the major axis of the TAD. With normalization, the mean barycenter distance between randomly distributed foci within the TAD is 0.71.

To assess the robustness of our method when performing BrdU experiments which had less localizations, we chose a TAD labeled by EdU for template (2547 localizations) to make a simulation. We randomly selected to leave half or quarter from them to test the robustness from our method. The mean value and standard deviation of *R*_*g*_ were as follows: all points (mean 160.3 nm); half points (mean 160.2 nm, std 1.5 nm); quarter points (mean 159.6 nm, std 2.2 nm). The mean value and standard deviation of center position were as follows: all points (mean (1937.9 nm, 1881.8 nm)); half points (mean (1937.4 nm, 1882.3 nm), std (1.3 nm, 2.5 nm)); quarter points (mean (1937.0 nm, 1881.7 nm), std (2.7 nm, 4.6 nm)). Each calculation was under the statistics from 30 tests.

For DBSCAN analysis, DBSCAN clustering was performed using custom Python code. In brief, for each localization, we first calculated the total localization number *N* within a threshold distance *r* from it. We set these two thresholds based on the total localization number from each image. Localizations were labeled as “core point” if their value of *N* were above the threshold. The “core points” were then clustered when their distance was under the reference distance *r*_*ref*_. Then, for each cluster, “border points” were defined as other points inside the radius *r*_*ref*_ from the “core points,” Finally, all points from each cluster were defined and saved for further analysis. The barycenter of each cluster was calculated by averaging the coordinates of all points. The radius of gyration (*R*_*g*_) was calculated as follows:

$$ {R}_g^2=\frac{1}{N}\ \sum \limits_{i=1}^N{\left({r}_i-\overline{r}\right)}^2 $$

In this formula,$\overline{r}$ is the centroid of all *N* localizations and *r*_*i*_ is the vector of an individual localization.

The radial distribution function (RDF) is a physical parameter describing the radial statistical probability distribution, which is defined as follows:

$$ {\int}_0^{\infty }{r}^2 dr\ {\int}_{-\pi /2}^{\pi /2}\cos \theta d\theta\ {\int}_0^{2\pi } d\varphi \ast {F}_{RDF}\left(r,\theta, \varphi \right)=1 $$

where *r* is the radial distance away from a given center and *F*_*RDF*_*(r)* represents the RDF. Under the above definition, *F*_*RDF*_(*r*, *θ*, *φ*) denotes the RDF in polar coordinates, which represents the distribution of the replication initiation tendency along the radius from a TAD. We made the assumption that there is no cell polarity factor that influences the distribution of replication initiations, such that, *F*_*RDF*_(*r*, *θ*, *φ*) could be denoted as *F*_*RDF*_(*r*).

For a simple comparison, we calculated the mean value from each TAD and normalized it by its radius of gyration (*R*_*g*_):

$$ RDD=\frac{mean\left({F}_{RDF}(r)\right)}{R_g} $$

where the radial density distribution (*RDD*) is the physical parameter used for comparison in the figures.

### Statistical analysis

All statistical analyses were performed using GraphPad Prism software version 6. Significance was analyzed by an un-paired two sample parametric t test: ****P < 0.0001, ***P < 0.0005, **P < 0.01, *P < 0.05; N.S., not significant. For scatter plots, horizontal lines and error bars represent the mean values ± s.d. For box plots, the center line indicates median number; box limits, 25% and 75% of the entire population; whiskers, observations within 1.5× the interquartile range of the box limits.

## Supplementary Information

**Additional file 1: Supplementary figures.**

**Additional file 2: Table S1.** Sequence of TADs and origins (TAD1 & TAD2).

**Additional file 3: Table S2.** Sequence of TADs and origins (TAD3 & TAD4 & TAD5).

**Additional file 4: Table S3.** Information for probes and primers.

**Additional file 5.** Review history.

## Data Availability

The localization coordinates used to generate the STORM images in Figs.1, 2, 3, and 4 and figures in additional files and used for statistic results as well as all codes have been deposited in Zenodo [84]. They are also available from the corresponding author Y.S. (sun_yujie@pku.edu.cn) on reasonable request.
