# Supplementary material for: Transcription-coupled structural dynamics of topologically associating domains regulate replication origin efficiency
Source: Genome Biol. 2021 Jul 12;22:206. doi: 10.1186/s13059-021-02424-w (PMC8276456; doi:10.1186/s13059-021-02424-w)
Supplement: Supplementary file 1 — Additional file 1: Supplementary figures. [file 13059_2021_2424_MOESM1_ESM.docx]

**Transcription-coupled structural dynamics of topologically associating domains regulate replication origin efficiency**

Yongzheng Li^1,6#^, Boxin Xue^1,7#^, Mengling Zhang^1#^, Liwei Zhang^2^, Yingping Hou^9^, Yizhi Qin^1^, Haizhen Long^2^, Qian Peter Su^1,3^, Yao Wang^1^, Xiaodong Guan^1^, Yanyan Jin^4^, Yuan Cao^1^, Guohong Li^2,5^, Yujie Sun^1,8*^

*^1^State Key Laboratory of Membrane Biology, Biomedical Pioneer Innovation Center (BIOPIC), School of Life Sciences, Peking University, Beijing, China 100871*

*^2^National Laboratory of Biomacromolecules, CAS Center for Excellence in Biomacromolecules, Institute of Biophysics, Chinese Academy of Sciences, Beijing, China 100101*

*^3^School of Biomedical Engineering, Faculty of Engineering and Information Technology, University of Technology Sydney, Sydney, NSW 2007, Australia.*

*^4^Department of Neurobiology, Beijing Centre of Neural Regeneration and Repair, Capital Medical University, Beijing, China 100101*

^5^*University of Chinese Academy of Sciences, Beijing, China 100049*

^6^*Academy for Advanced Interdisciplinary Studies, Peking University, Beijing, China 100871*

^7^*College of Chemistry and Molecular Engineering, Peking University, Beijing, China 100871*

*^8^College of Future Technology, Peking University, Beijing, China 100871*

*^9^Peking-Tsinghua Center for Life Sciences, Academy for Advanced Interdisciplinary Studies, Peking University, Beijing 100871, China*

*# These authors contribute to the work equally.*

^*^*Corresponding author*

^*^ Email: sun_yujie@pku.edu.cn

**Supplementary Figures and Figure Legends**


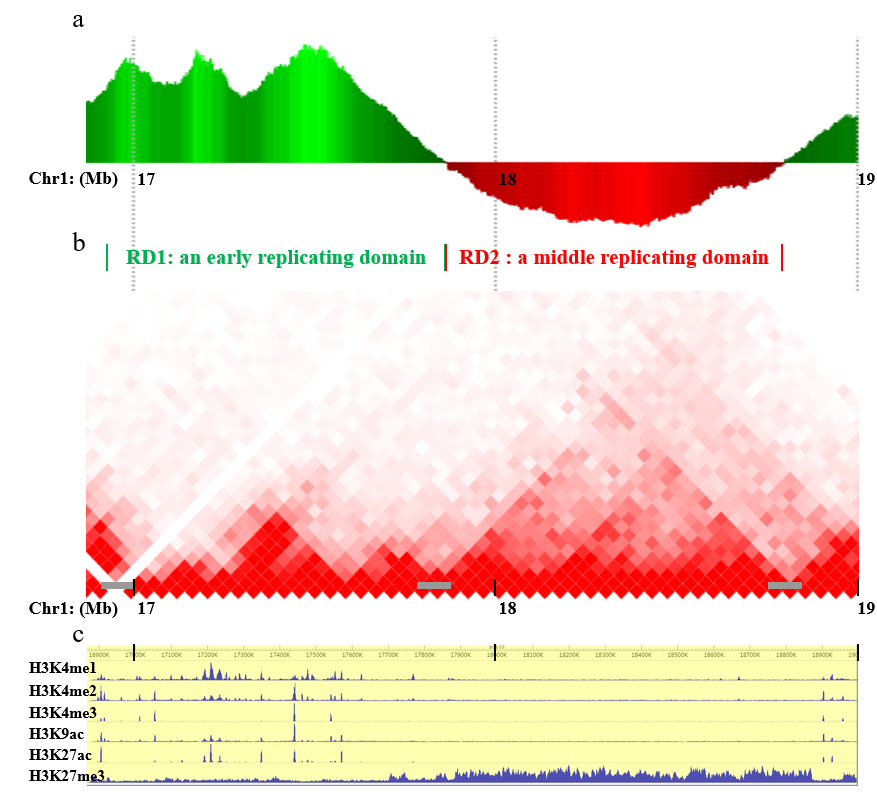


**Figure S1 Identification and histone modifications of two TADs from the replication timing profile and Hi-C interaction heatmap of HeLa cells. a-b,** Depiction of the replication timing profile (green peaks for early RDs and red peaks for middle or late RDs) and Hi-C interaction heatmap in the same genomic region of chromosome 1. The replication timing profile was obtained from the Replication Domain Genome Browser of the Gilbert lab ([https://www2.replicationdomain.com/ genome_browser](https://www2.replicationdomain.com/%20genome_browser)). The Hi-C interaction heatmap was obtained from ENCSR693GXU. Grey bars: TAD boundaries. (Methods) Two TADs were selected. TAD1: an early replicating domain (Chr1:16911932-17714928). TAD2: a middle replicating domain (Chr1:17722716-18846245). **c,** Profiles of histone modifications are from public data hubs (ENCODE data portal) of WashU Epigenome Browser.


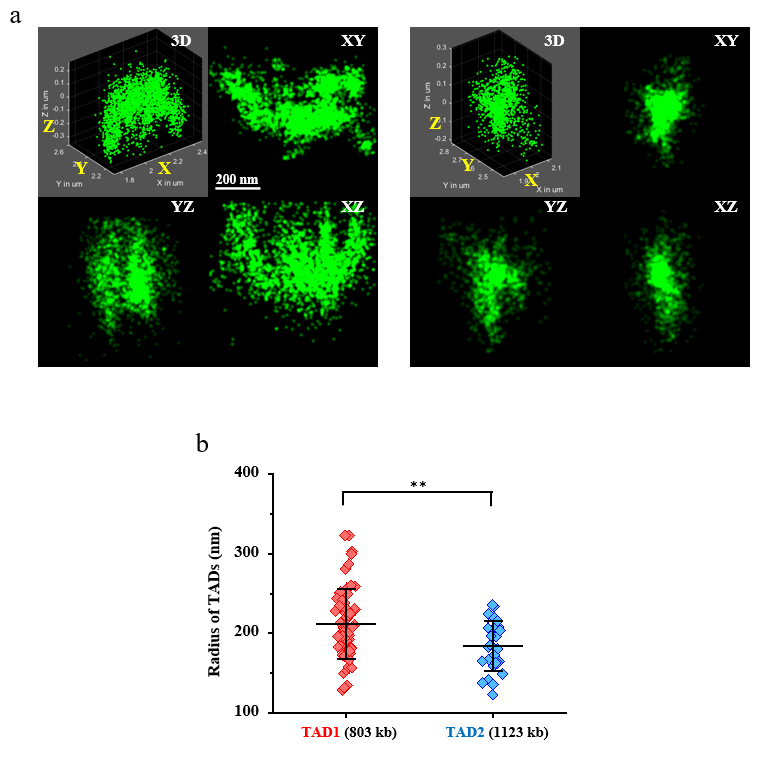


**Figure S2. 3D visualization and radii of TAD1 and TAD2.** The definitions and labeling procedures of TADs and origins are identical with those in Figure 2. **a**, Representative 3D STORM images of TADs. One 3D presentation with 3 projected images. **b,** 3D radius of gyration of TAD1 and TAD2. For lines and statistics in **b** see the description in the legend of Figure 1 (n ≥20 cells).


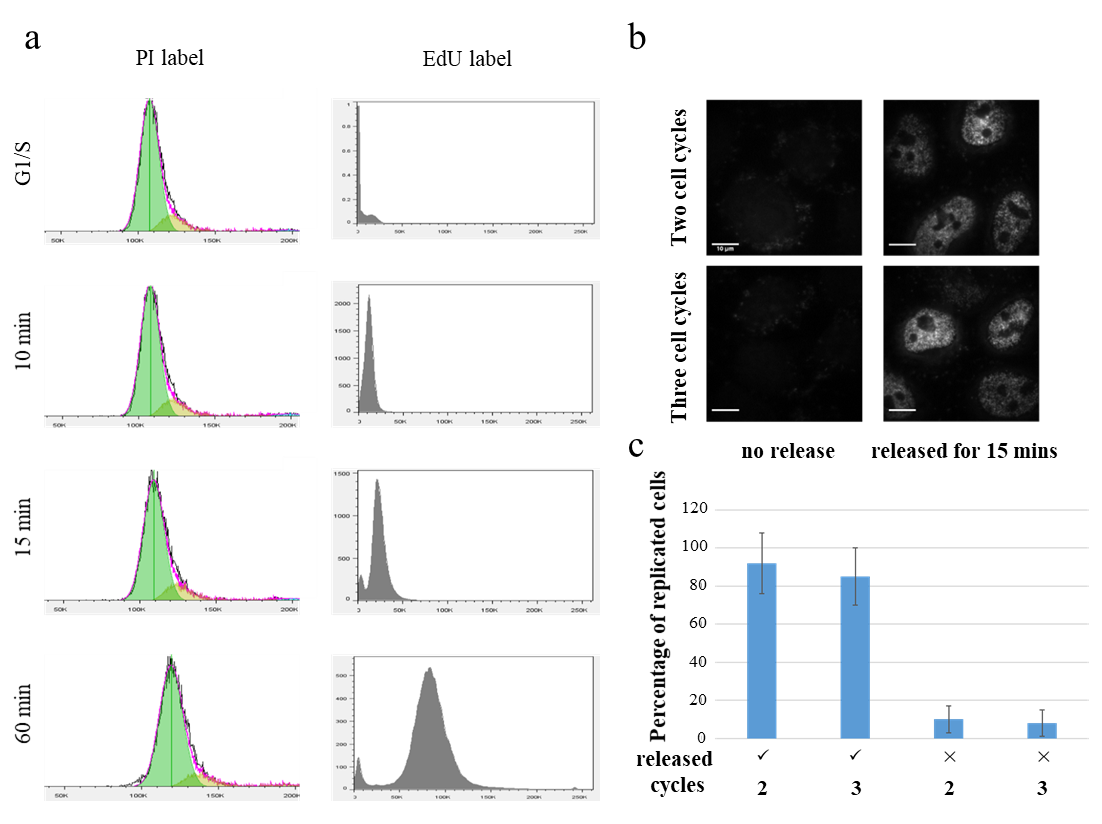


**Figure S3. Quantification of cell synchronization by EdU labeling.** **a**, Left panel, cells were synchronized for two cell cycles and then labeled by PI for the total DNA at G1/S transition or released into S phase for 10, 15 or 60 minutes. Flow cytometry data of PI labeling did not suffice to indicate the replicated DNA as only a small portion of the total DNA was replicated during the first 15 minutes in the S phase. To improve the resolution of labeling replicated DNA, cells were released into the S phase and simultaneously labeled by EdU for indicated time. The significantly increased fluorescence of EdU (right panel) shows that the cell synchronization minimally impacts the replication of cells. **b**, After two (upper) or three (lower) cycles of synchronization, cells were synchronized to the G1/S transition. EdU were added when the synchronized cells were released (right) for 15 min or were not released (left). **c**, percentage of the replicated cells in **b**. Replicated cells were defined by the three folds of the fluorescence of the nucleus to the background (3 replicates, 200 cells for each group). EdU labeling showed more than 80% cells entering the S phase, similar with the previous work [25].


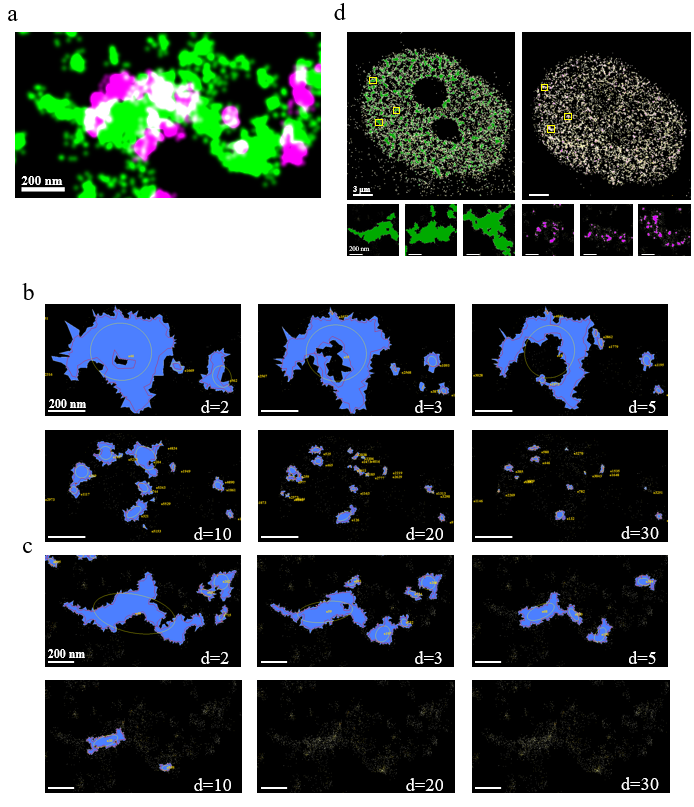


**Figure S4. Quantification of the density factor in SR-Tesseler analysis. a,** Dual-color STORM imaging of TADs (also early replicating domains, green) and origins (purple) as in Fig. 1c. **b** and **c,** Analysis of TADs and origins by SR-Tesseler with density factors from 2 to 30. **b**, Origins close to each other in a TAD could not be separated when the density factor was set from 2 to 10. Origins were too small or even missing when the density factor was set to 30. When the density factor was set to 20, approximately 5,000 origins were clearly identified at the beginning of the S phase in one cell, similar with a previous report [21]. **c**, TADs close to each other could not be separated when the density factor was set to 2. TADs were too small or even missing when the density factor was set from 5 to 30. When the density factor was set to 3, approximately 700 TADs were clearly identified, similar with a previous report [7]. Therefore, density factor was set to 3 for analyzing TADs and 20 for analyzing RFi. **d**, TADs (green) and origins (purple) identified by SR-Tesseler from the STORM images using a density factor of 3 for TADs and a density factor of 20 for origins. The areas inside the yellow squares are shown at higher magnification below each nucleus.


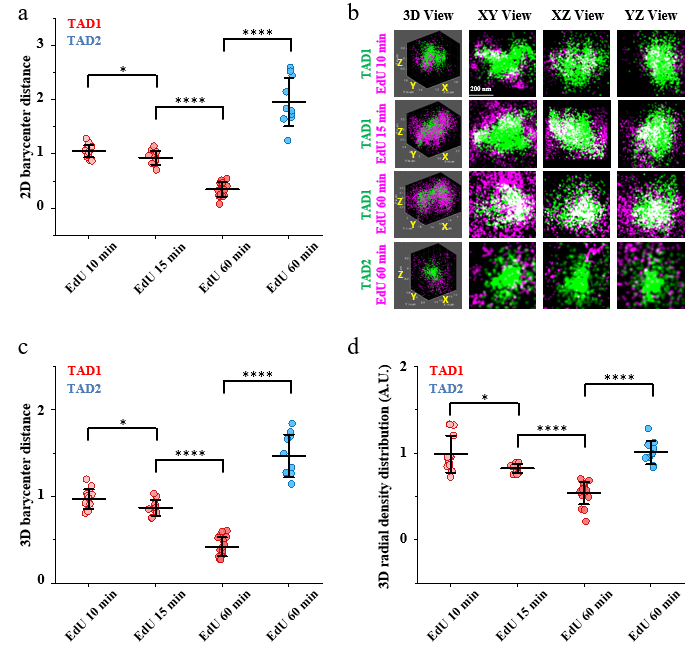


**Figure S5. Replication patterns of TADs in the S phase as determined by DBSCAN. a,** 2D barycenter distances between the TADs and their spatially associated RFi as determined by DBSCAN (Methods) in Figure 1**a**. **b,** Representative 3D STORM images of TAD1 and TAD2 labeled by Oligopaint probes (green) and RFi labeled metabolically for different durations (purple). Metabolic labeling of DNA replication was performed by supplying EdU to the cells upon release into the S phase for 10 min, 15 min, and 60 min (purple). **c,** 3D barycenter distances between the TADs and their spatially associated RFi as determined by DBSCAN in **b**. **d,** Radial density distribution of the RFi in TADs as determined by DBSCAN (Methods) in **b**. For lines and statistics in **a**, **c**, and **d** see the description in the legend of Figure 1 (n ≥10 cells).


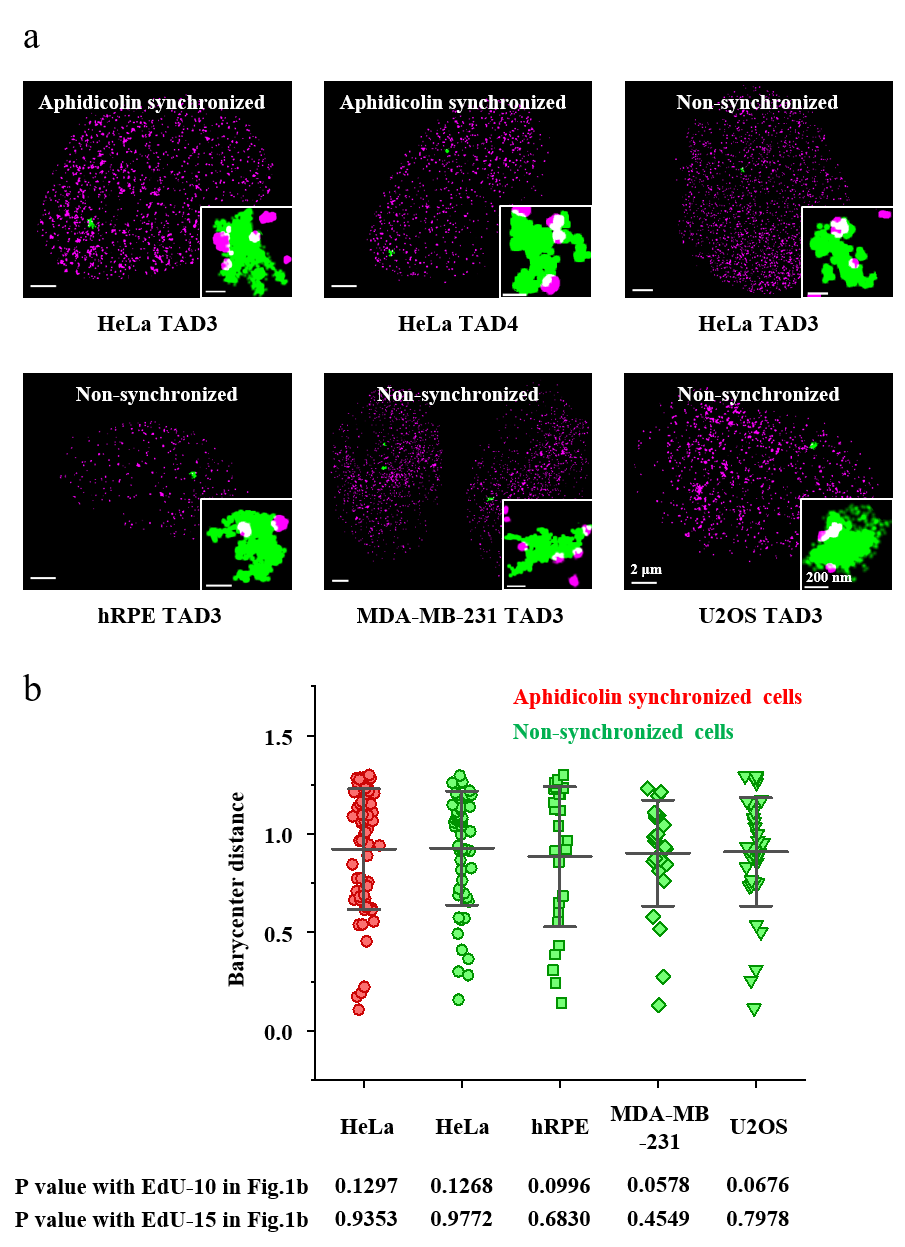


**Figure S6 Super-resolution imaging of replication initiation sites and TADs in the S phase in synchronized or non-synchronized cells of different cell lines. a**, Representative STORM images of TAD3 and TAD4 (Additional file 2: Table S2) labeled by Oligopaint probes (green) and early replication initiation sites metabolically labeled by EdU (purple) (Methods). The strategy of identifying replication initiating cells in non-synchronized cells is described in Figure S7. The areas inside the yellow squares are shown at higher magnification to the bottom right of each nucleus. Portions of the two signals that overlap are shown in white. **b**, Barycenter distances between replication initiation sites and TADs of different cell lines in **a** (n ≥10 cells). Barycenter distances of replication initiation sites in TAD3 and TAD4 were combined. P value showed that spatial distribution of replication initiation sites in non-synchronized cells were close to that in aphidicolin synchronized cells as shown in Figure 1. For lines and statistics in **b** see the description in the legend of Figure 1 (n ≥10 cells).

**
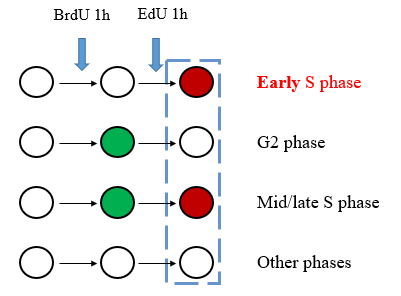
**

**Figure S7. Strategy of identifying cells in the early S phase among non-synchronized cells.** Cells were labeled by BrdU for 1 hour, followed by 1 hour EdU labeling and fixation. As BrdU and EdU can only be globally incorporated into DNA during S phase, this strategy allows to identify the cell cycle stage of a cell based on its fluorescence color. For cells fixed in early S phase (cells with only red fluorescence), we referred to typical patterns of replication foci (Fig. S6a) to select cells that had entered and been EdU-labeled for nearly 15 minutes.


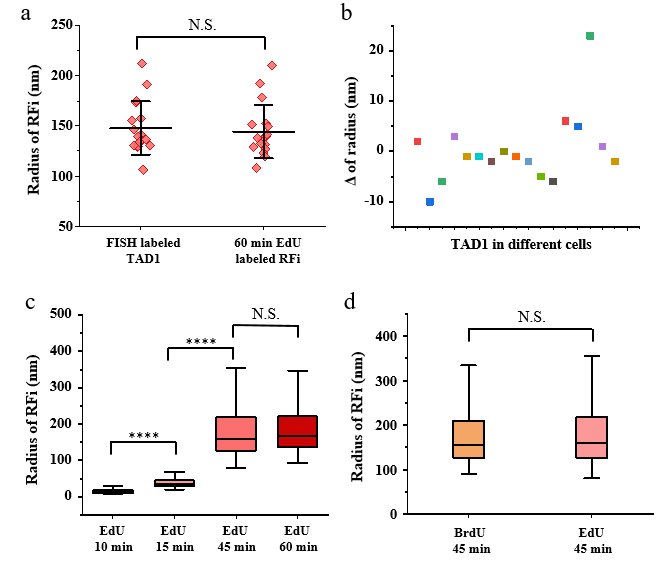


**Figure S8. Quantitative characterization of metabolically labeled RFi. a-b,** Comparison of FISH-labeled TAD1 and co-localized 60-min EdU labeled RFi. **a**, Radii of FISH-labeled TAD1 and 60-min EdU labeled RFi. **b**, Changes in radius between FISH-labeled TAD1 and its corresponding 60-min EdU labeled RFi in the same cell. Different colors represent different cells. **c**, Radii of RFi labeled for 10 min, 15 min, 45 min and 60 min upon release into the S phase. **d**, Radii of RFi labeled for 45 min by BrdU (yellow) or EdU (pink) at the beginning of the S phase. For lines and statistics in **a**, **c**, and **d** see the description in the legend of Figure 1 (n =10 cells).


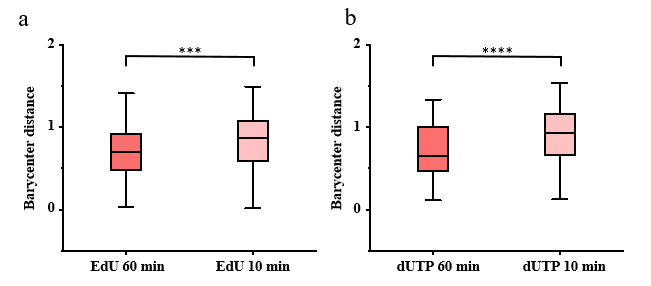


**Figure S9. Comparison of RFi labeled by different metabolic labeling methods and for different durations. a,** Box plot of barycenter distances between BrdU-labeled RFi and EdU-labeled RFi. BrdU was supplied for 45 min upon release into the S phase, whereas EdU was supplied for 10 or 60 min. **b,** Box plot of barycenter distances between EdU-labeled RFi and dUTP-atto550 labeled RFi. EdU was supplied for 45 min upon release into the S phase, whereas dUTP was supplied for 10 or 60 min. For lines and statistics in **a** and **b** see the description in the legend of Figure 1 (n =10 cells).


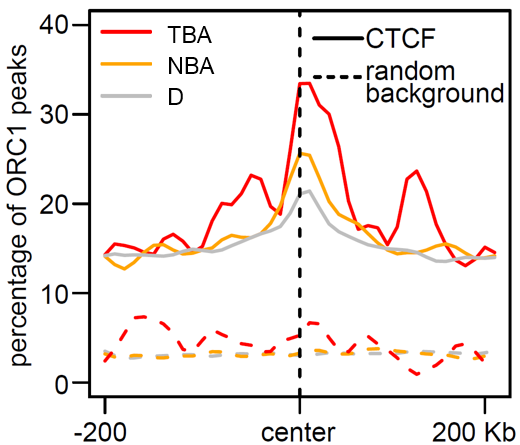


**Figure S10. Co-localization of replication origins with CTCF-cohesin binding sites.** Histograms of distance between CTCF-cohesin binding sites and replication origins are presented in solid lines; those for randomly-selected sites are presented by dotted line. TBA (TAD boundary active origins): red lines; NBA (Non-TAD boundary active origins): yellow lines; D (dormant replication origins): grey lines. Center dashed line is the sites with overlapped binding of CTCF and cohesin.


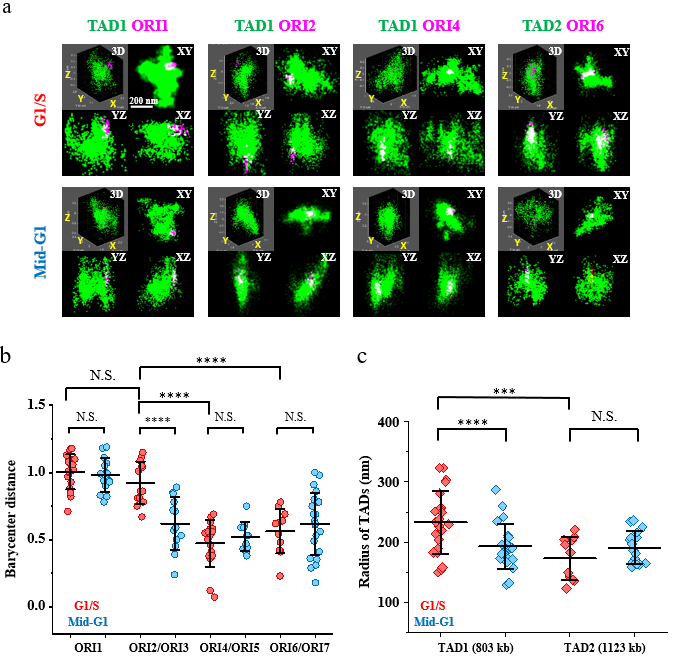


**Figure S11. 3D distribution of replication origins in TADs in the G1 and G1/S phase.** The definitions and labeling procedures of TADs and origins are identical with those in Figure 2. **a**, Representative 3D STORM images of TADs (green) and their origins (purple) in the G1 and G1/S phases. Upper row: TADs and origins labeled at the G1/S transition. Lower row: TADs and origins labeled approximately 5 hours into the G1 phase. **b**, 3D Barycenter distances between all 7 origins and the 2 related TADs in a. **c,** 3D radius of gyration of TAD1 and TAD2 in the G1 and G1/S phases. For lines and statistics in **b** and **c** see the description in the legend of Figure 1 (n ≥10 cells).


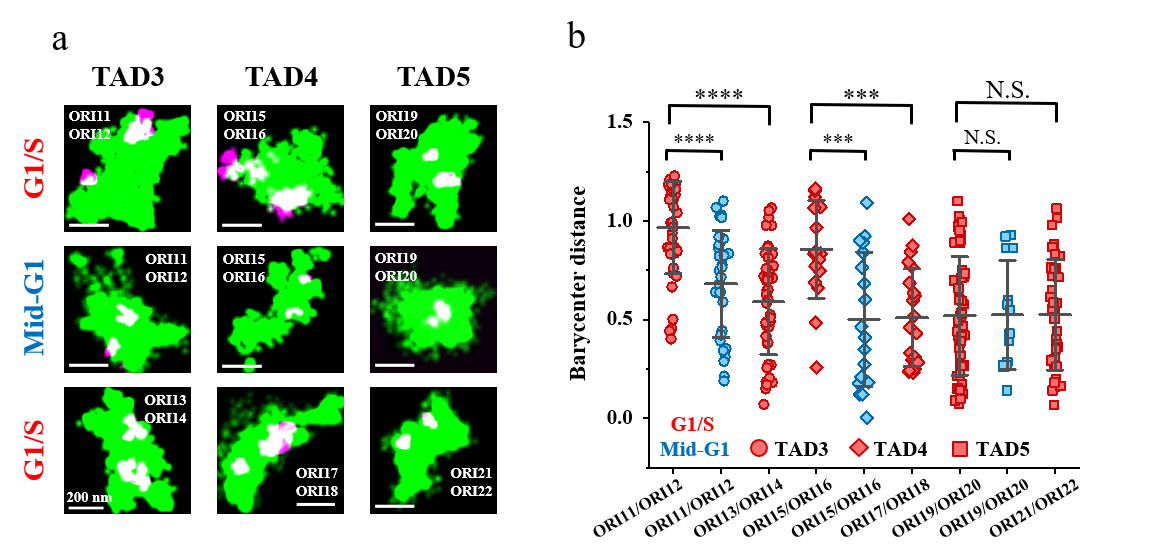


**Figure S12. Spatial distribution of replication origins in TAD3, TAD4 and TAD5 in the G1 and G1/S phases. a,** Representative STORM images of TADs (green, including TAD3, TAD4 and TAD5) and their origins (purple) labeled by FISH with oligoprobes in the G1 and G1/S phases. Top, TADs and active origins labeled at the G1/S transition. Middle, TADs and origins labeled at approximately 5 hours into the G1 phase. Bottom, TADs and dormant origins labeled at the G1/S transition. Portions of the two signals that overlap are shown in white. **b,** Barycenter distances between origins and TADs in **a** (n ≥10 cells). For lines and statistics in **b** and **c** see the description in the legend of Figure 1.


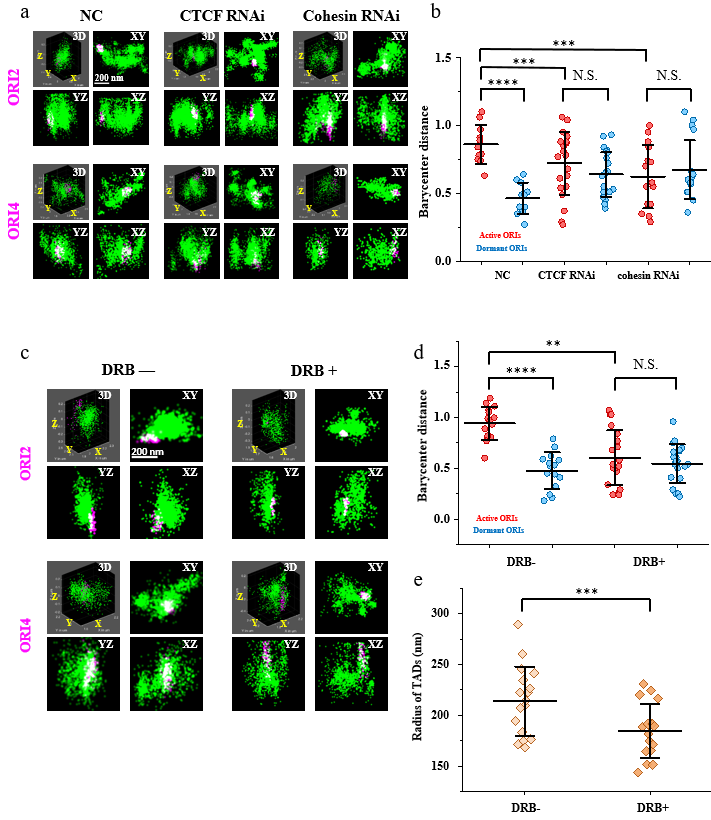


**Figure S13. 3D Distribution of replication origins in TAD1 with transcription elongation inhibition or down-regulation of CTCF or cohesin. a**, Representative 3D STORM images of origins (purple) in TAD1 (green) after treatment of cells with the indicated siRNAs. **b**, 3D barycenter distances between active (ORI2 and ORI3) or dormant (ORI4 and ORI5) origins in TAD1 after treatment of cells with the indicated siRNAs as in **a**. **c**, Representative 3D STORM images of origins (purple) in TAD1 (green). Restricted by the space, only ORI2 and ORI4 are shown. Left: no DRB. Right: with DRB. **d,** 3D barycenter distances between active (ORI2 and ORI3) and dormant (ORI4 and ORI5) origins in TAD1 with or without DRB treatments. **e**, 3D radius of gyration of TAD1 treated with or without DRB. For lines and statistics in **b**, **d**, and **e** see the description in the legend of Figure 1 (n ≥10 cells).


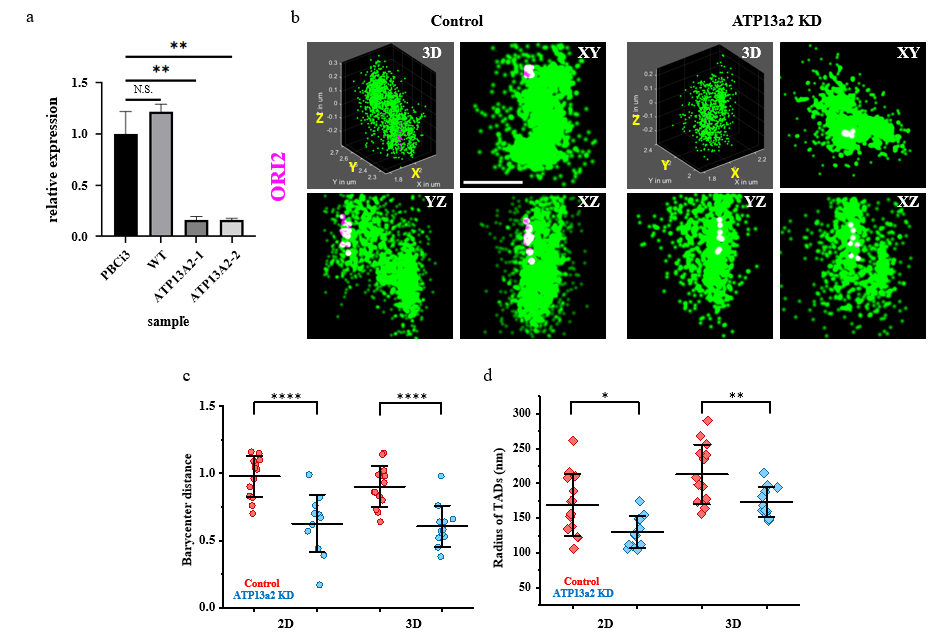


**Figure S14. 3D Distribution of ORI2 in RD1 with inhibiting transcription elongation of *ATP13a2* by CRISPRi. a,** Efficiency of CRISPRi (n=3 replicates) **b**, Representative 3D STORM images of ORI2 (purple) in TAD1 (green) after knockdown of *ATP13a2* by CRISPRi. **c**, 2D or 3D barycenter distances of ORI2 in TAD1 with or without CRISPRi. **d**, 2D or 3D radius of TAD1 with or without CRISPRi (n ≥10 cells). For lines and statistics in **a**, **c**, and **d** see the description in the legend of Figure 1 (n ≥10 cells).


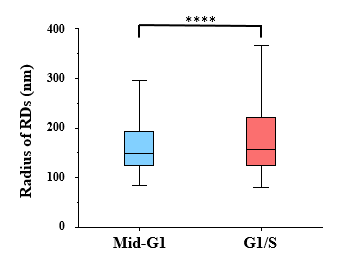


**Figure S15. Radii of metabolically labeled TADs in the G1 and G1/S phase.** TADs were labeled by EdU for 45 min upon release into the S phase. In the next cell cycle, cells were fixed in the mid-G1 or G1/S phase. For lines and statistics see the description in the legend of Figure 1 (n =10 cells).


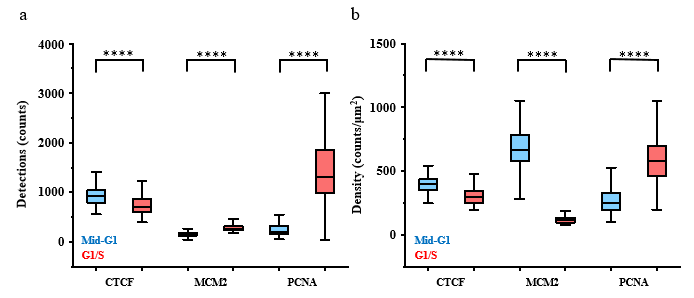


**Figure S16. Single molecule detection counts and density of CTCT, MCM2, and PCNA in early replicating TADs in the mid-G1 and G1/S phases.** Counts are shown in **a** and molecule density is shown in **b**. The reduced number of single-molecule CTCF detections and molecule density in the CTCF foci indicate that CTCF molecules dissociate from chromatin in the G1 phase. The increased number of single-molecule MCM2 detections and decreased molecule density in the MCM2 foci indicate gradual association of MCM2 with chromatin and dislocation from DNA. The increased number of single-molecule PCNA detections and molecule density in the PCNA foci indicate assembly of replication factories in the G1 phase. See details in the main text. For lines and statistics see the description in the legend of Figure 1 (n =10 cells).
