# Supplementary material for: Transcription-coupled structural dynamics of topologically associating domains regulate replication origin efficiency
Source: Genome Biol. 2021 Jul 12;22:206. doi: 10.1186/s13059-021-02424-w (PMC8276456; doi:10.1186/s13059-021-02424-w)
Supplement: Supplementary file 5 — Additional file 5. Review history. [file 13059_2021_2424_MOESM5_ESM.docx]

**1^st^ round**

**Reviewer 1**

In this manuscript, Dr. Yujie Sun's group uses super-resolution microscopy to resolve the localization dynamics of origin proximal sequences and replication factors with topologically associated domains (TADS). A technical advance was the adaptation of oligopaint and STORM imaging (OLIGOSTORM) to label specific TADs, their boundaries, or origin proximal sequences in order to study nuclear architecture during G1 and S phase at super resolution. The authors find that early/efficient replication foci initiate at the periphery of the TAD and move inwards by 60 minutes. They also find that TADs reorganize between G1 and S phase, with origin proximal sequences relocating to the periphery of the tad and that cohesin and ctcf contribute to this reorganization. I am not an expert in super-resolution imaging, but I did find the data compelling and seemingly well controlled. The authors found that the Mcm2-7 helicase (which is loaded in G1) starts off at the periphery of the TAD, but by the G1/S transition it appears to be localized throughout the TAD which is consistent with the licensing of excess origins. Finally PCNA remains at the boundaries of the TAD suggesting that active replication occurs at the periphery. This leads to a model where there is active re-organization of the chromatin during G1 which leads to active/efficient origins moving to the periphery for initiation.

My only concern with the work is that it uses an aphidicolin arrest and release to capture early initiation events. The concern here is that the intra S Phase checkpoint is being activated which prevents the activation of later inefficient origins.. It would be great if the authors could use cell sorting or some other method to enrich cells in G1 and early S-phase so as to confirm their results (origin mobilization/dynamics within the TAD) in a normal cell cycle.

**Reviewer 2**

Reviewer's comments on the manuscript entitled Transcription-coupled structural dynamics of topologically associating domains regulate replication origin efficiency' authored by Sun and colleagues

Eukaryotes replicate their genome in a spatially and temporally defined manner. Work from Gilbert lab has shown that topologically associated domains (TADs) are stable units of replication timing regulation. Further, the replication domain boundaries were found to correlate with TAD boundaries. Most of the work studying the correlation of TADs and replication timing has been based on genomic approaches. A recent manuscript in Biorxiv showed a role to transcription within TAD boundaries in promoting replication origin firing (Lombardi and Tarsounas, 2021).

In the current manuscript, the authors use super resolution STORM imaging to map the spatial organization of replication origins relative to TADs. They report that origins that are highly efficient or low efficient localize homogeneously within TADs during G1, however, the highly efficient origins relocalize to the periphery of the TADs during S-phase. This relocation is dependent on CTCF and Cohesin. This is a natural extension of the nice work published by the group in PNAS using STORM imaging demonstrating that spatiotemporal dynamics of the replication domains is mediated by CTCF-organized chromatin domains.

This is an interesting report, but there are many technical issues that need to be addressed:

1) The authors use a 10-min pulse labeling of the replicating DNA by supplying thymidine analog EdU to look at replication domains. It may be worth noting that work from Belmont and colleagues has shown that '…CHO cells showed that newly replicated DNA exits replication foci after an ∼9-min pulse.' It is important to optimize the pulse labeling duration in each cell line to ensure that what we are looking at is indeed 'replication foci'. It is inaccurate to call these sites as replication initiation sites, because the fork would have progressed significantly in 10 minutes. Shorter pulse would need to be used to substantiate their claims.

2) Cell synchronization methods using drug (thymidine followed by aphidicolin etc) may not be the best methodology to use. Aphidicolin may cause replication stress and it can alter chromatin architecture (as also acknowledged by the authors). The best synchronization procedures include 'cell elutriation' techniques.

3) The authors describe that the replication foci (based on the 10 min pulse, that they presume includes an origin) is at the periphery in TAD1 (early replicating domain) based on 10 and 15 min pulse experiments. There is no control for 10-15 minutes pulse for TAD2 (late replicating), only 60 mins is shown.

4) All the conclusions of the manuscript are based on one TAD that is an early replicating domain and another one that is late replicating. Is this true globally? At least three examples need to be shown, each for early domain and late domain. Also, all the conclusions are drawn from a single cell line HeLa. It would be valuable to understand if similar organization is valid in different cell types, especially the non-cancerous ones.

5) The data do not support the conclusion that 'We obtained a global view of the spatial distribution of replication initiation sites relative to early RDs in a cell. Analysis of the dual-color STORM images showed that there were averagely 7 replication initiation sites in each RD.'

6) The correlation of different ORIs (based on Orc1 and H2AZ ChIP data) and TAD positioning was convincing and a strong data in support of the author's conclusions. It would be nice to use other examples to confirm that this holds true genome-wide.

7) For figure 3, where CTCF and Cohesin siRNAs have been performed, it would be good to have a control (For example, condensin siRNA).

8) The authors note: 'Taken together, these data revealed that the structural re-organization within the TADs and decompaction in the G1 phase facilitate the relocation of highly-efficient origins from the TAD interior to the periphery'. What is the advantage of initiating at the periphery? It would be good to include that in the discussion.

9) 'Upon down-regulation of CTCF or cohesin using RNAi (Figure 3a insets, Figure 3b, left panel), we found that the highly efficient origins (ORI2 and ORI3) were not relocated to the TAD periphery at the G1/S transition in both 2D and 3D images.' Did this lead to change in the timing of DNA replication? What was the consequence?

10) A recent study (as noted above) pointed that 'early constitutive origins fire most frequently within or adjacent to TAD boundaries genome-wide'. And it was proposed: 'active transcription emanating from CTCF binding sites displaced replication initiation sites towards the edges of the TAD boundary.' The observations made by the current paper also support the view that relocation of replication origins is dependent on chromatin-looping, however there are key differences that need clarification.

11) It is known that without cohesin, mammalian TAD-like domains can form spontaneously by chromatin interactions. Does this impact the replication within these kinds of TADs?

12) The transcription inhibition experiments are interesting, but do not address some concerns. For example, inhibiting transcription in the fly has been shown to cause reorganization of TAD structure, and a weakening of border strength. Further, Cavalli's group has shown that transcription by itself is insufficient and that transcription factors may insulate CTCF-independent TAD borders. Also, using STORM imaging, Cavalli group demonstrated that within TADs active domains were found at the borders of repressed ones. The authors in this manuscript suggest, 'that transcription-dependent chromatin structural re-organization within the TADs exposes a subset of origins to the physical boundary of a TAD, which are preferentially used for replication initiation.' Recent work shows that when transcription is inhibited chemically or deregulated by oncogene overexpression, replication origins become repositioned to the centre of the TAD. It may be important to discuss these studies in the current manuscript and discuss the caveats of the transcription inhibition experiments.

13) The data using MCM and PCNA is not convincing. PCNA is found in soluble nucleoplasmic pool and a preextraction procedure is critical to look at the chromatin-associated form. No such methodology has been used (as per details provided in the methods section). Further, the authors conclude that PCNA foci/clusters at the TAD boundary is the reason why origins at TAD periphery get activated. Detailed experiments are needed to support this claim.

14) Data suffers from overinterpretation: 'The CRISI model suggests that the spatial localization of an origin in a TAD determines its replication efficiency.' There are no experiments supporting efficiency of replication.

**Reviewer 3**

This manuscript presented a study that revealed a new mechanism for selecting replication origin using two-color STORM imaging to map the spatial distribution of replication origins in the context of topologically associating domains (TAD). They labeled two specific TAD sites (one enriched in early replicating domain and one enriched in mid-replicating domain) and used pulse labeling of EdU and BrdU to compare the spatial co-localization between these two TAD sites and replication origins in G1 and G1/S phase. They reported that replication origins initiated separately at the periphery of a TAD. They found that chromatin reorganization within the TADs in G1 phase, which is driven by disruption of chromatin loops during transcription elongation. The overall studies are rigorous and thorough. The findings are interesting to biologists in the field of DNA replication and genome organization. I recommend the publication of this manuscript after minor revision.

The authors used BrdU and EdU labeling for either replication origins or replication domains. BrdU is based on antibody labeling, which often suffers from much lower labeling density compared to click chemistry-based labeling. How did the authors ensure the unbiased comparison?

In Methods, the identification of barycenter is a crucial factor in this study, but how the barycenter is defined here was not clearly described. As barycenter involves the calculation of cluster mass, how was "mass" defined in STORM images was not clear. In line 424 in Supplementary Materials, the authors stated "Different density factors were evaluated for identification of TADs and small foci such as protein clusters or replication origins". This description was too vague.

**Authors’ response**

1) Regarding the major concerns of Referees 1 and 2 on the use of aphidicolin to synchronize cells and capture early replication initiation events, we conceived a strategy and performed new experiments to label replication initiation sites at the early S phase without cell synchronization. Details of the strategy can be found in “point-by-point response” as well as in Fig. S6. The new results obtained in non-synchronized cells also showed that replication initiates at the periphery of TADs at the early S phase (Fig. S7), consistent with that obtained in aphidicolin-synchronized cells (Fig. 1a).
2) Regarding the concern that “all the conclusions are drawn from a single TAD or in a single cell line”, we conducted new experiments in additional TADs and cell lines to strengthen the conclusions of our work (Fig. S12). These new experiments conducted on additional TADs and cell lines helped to validate the conclusions obtained in the original manuscript.

**Point-by-point response to Reviewers’ comments:**

**Reviewer #1:**

*In this manuscript, Dr. Yujie Sun's group uses super-resolution microscopy to resolve the localization dynamics of origin proximal sequences and replication factors with topologically associated domains (TADS). A technical advance was the adaptation of oligopaint and STORM imaging (OLIGOSTORM) to label specific TADs, their boundaries, or origin proximal sequences in order to study nuclear architecture during G1 and S phase at super resolution. The authors find that early/efficient replication foci initiate at the periphery of the TAD and move inwards by 60 minutes. They also find that TADs reorganize between G1 and S phase, with origin proximal sequences relocating to the periphery of the tad and that cohesin and ctcf contribute to this reorganization. I am not an expert in super-resolution imaging, but I did find the data compelling and seemingly well controlled. The authors found that the Mcm2-7 helicase (which is loaded in G1) starts off at the periphery of the TAD, but by the G1/S transition it appears to be localized throughout the TAD which is consistent with the licensing of excess origins. Finally, PCNA remains at the boundaries of the TAD suggesting that active replication occurs at the periphery. This leads to a model where there is active re-organization of the chromatin during G1 which leads to active/efficient origins moving to the periphery for initiation.*

**Author reply:**

We are very grateful to the reviewer for his/her appreciation of our technical strategy to apply super-resolution imaging in the study of replication origins. Replication origin activation in mammalian cells has been long under debate as many genetic and epigenetic features are found to be related to the efficiency of replication origins. Using super-resolution imaging techniques to map replication origins within individual replication domains, we were able to investigate this question from a unique perspective and link replication origin activation to chromatin 3D structure. Together with the literatures reporting the correspondence between chromatin 3D structure and genetic/epigenetic features, our finding reconciles existing replication origin activation models that are based on various genetic codes and epigenetic modifications and represents a conceptual advance in this field.

*My only concern with the work is that it uses an aphidicolin arrest and release to capture early initiation events.  The concern here is that the* *intra S Phase checkpoint is being activated which prevents the activation of later inefficient origins.  It would be great if the authors could use* *cell sorting or some other method to enrich cells in G1 and early S-phase so as to confirm their results (origin mobilization/dynamics within the TAD) in a normal cell cycle.*

**Author reply:**

We thank the reviewer for raising the concern that using aphidicolin to synchronize cells can lead to intra S phase checkpoint activation, which may question the validity of our conclusions. Practically, as replication initiation takes place in a very short time window, we synchronized cells to the G1/S boundary to increase the probability for finding replication initiation events under the microscope. To eliminate the possibility that the aphidicolin treatment may artificially lead to our observations, we followed the reviewer’s suggestion and designed experiments to observe replication initiation sites at the early S phase without cell cycle synchronization. To do so, we applied BrdU labeling (green) for 1 hour, followed by 1 hour EdU labeling (red) and fixation of living cells cultured in a petri dish. As BrdU and EdU can only be globally incorporated into DNA during S phase, this strategy allows to identify the cell cycle stage of a cell based on its fluorescence color (Fig. R1a). For cells fixed in early S phase (cells with only red fluorescence), we refer to typical patterns of replication foci reported by our team (Q. Su … Y. Sun. *PNAS,* 2020) and other teams (V. Chagin … M. Cardoso, *Nature Comm*, 2016) to select cells that had entered and been EdU-labeled for nearly 15 minutes like the purple punctate patterns shown in Figure R1b. By labeling TADs with FISH, we were able to measure the distribution of replication initiation sites relative to TADs in non-synchronized cells. We proved that replication initiates at the periphery of a TAD in non-synchronized cells (Fig. R1b), consistent with the observation in aphidicolin-treated cells (Fig. R1b&c and Fig. 1a). Besides HeLa cells, we also checked other cell lines including hRPE, U2OS and MDA-MB-231 and obtained the same results (Fig. R1b&c). In summary, these new experiments conducted on non-synchronized cells helped to validate the conclusions obtained on synchronized cells.

Figure R1. **a**, schematic illustration of the strategy for labeling and distinguishing non-synchronized cells in early S phase; **b&c**, Super-resolution imaging of replication initiation sites and TADs in the early S phase in synchronized or non-synchronized cells of different cell lines; P values show that spatial distribution of replication initiation sites in non-synchronized cells were close to that in aphidicolin-synchronized cells as shown in Figure 1. P values were obtained by un-paired two sample parametric t test.

**Reviewer #2:**

*Eukaryotes replicate their genome in a spatially and temporally defined manner. Work from Gilbert lab has shown that topologically associated domains (TADs) are stable units of replication timing regulation. Further, the replication domain boundaries were found to correlate with TAD boundaries. Most of the work studying the correlation of TADs and replication timing has been based on genomic approaches. A recent manuscript in Biorxiv showed a role to transcription within TAD boundaries in promoting replication origin firing (Lombardi and Tarsounas, 2021).*

*In the current manuscript, the authors use super resolution STORM imaging to map the spaeential organization of replication origins relative to TADs. They report that origins that are highly efficient or low efficient localize homogeneously within TADs during G1, however, the highly efficient origins relocalize to the periphery of the TADs during S-phase. This relocation is dependent on CTCF and Cohesin. This is a natural extension of the nice work published by the group in PNAS using STORM imaging demonstrating that spatiotemporal dynamics of the replication domains is mediated by CTCF-organized chromatin domains. This is an interesting report, but there are many technical issues that need to be addressed:*

**Author reply:**

We are very grateful to the reviewer for the overall positive comment on our work. We also appreciate that the reviewer pointed out the relevant work by Lombardi and Tarsounas recently published in *Biorxiv*. Replication origin activation in mammalian cells has been long under debate as many genetic and epigenetic features are found to be related to the efficiency of replication origins. Using super-resolution imaging techniques to map replication origins within individual replication domains, we were able to investigate this question from a unique perspective and link replication origin activation to chromatin 3D structure.

We also appreciate for the technical issues raised by the reviewer. As the first study to image replication origins within individual TADs using super-resolution imaging, we are supposed to thoroughly validate the methodologies used in the study. As detailed in the following point-by-point response, we have followed the reviewer’s instructions and greatly strengthened the technical validity of our work.

1) *The authors use a* *10-min pulse labeling of the replicating DNA by supplying thymidine analog EdU to look at replication domains. It may be worth noting that work from Belmont and colleagues has shown that '…CHO cells showed that newly replicated DNA exits replication foci after an ∼9-min pulse.'  It is important to* *optimize the pulse labeling duration in each cell line to ensure that what we are looking at is indeed 'replication foci'. It is inaccurate to call these sites as replication initiation sites, because the fork would have progressed significantly in 10 minutes. Shorter pulse would need to be used to substantiate their claims.*

**Author reply:**

We thank the reviewer for pointing out the nice work by Belmont and his colleagues (X. Deng …A.S. Belmont, *Curr Biol*, 2016). We note that while in principle the shorter the pulse-chase duration, the more precise replication initiation sites could be marked，the practical choice of labeling duration need to be considered based on multiple parameters, including labeling density, chromatin topology (usually described by radius of gyration, Rg) and spatial resolution. Firstly, like the EdU pulse-chase titration in the work by Belmont, we have also tried EdU titration of 5, 10 and 15 minutes. As EdU was added immediately after the release of replication arrest, majority of the pulse-labeled RFi were supposed to contain the corresponding replication initiation sites. We observed that 5 min EdU pulse-chase yielded weak and sparse fluorescence labeling, making it difficult to precisely locate the distributions of replication initiation sites. For 10 and 15 min EdU pulse-chase, we observed reduced barycenter distances (Fig. 1b) and increased radius of gyration (Fig. S8c) from 10 min to 15 min. Thus, among the three EdU labeling durations, 10 min pulse-chase is more proper to represent replication initiation sites. Secondly, based on the *in situ* DNA replication speed measured in our previous work (Su …Y.J.Sun. *PNAS* 2020), the length of replicated DNA would be between 0 and 20 kbp, dependent on when replication initiated during the 10 min pulse-chase duration, and the corresponding Rg would be between 0 and ~50 nm (B. Bintu…X. W. Zhuang. *Science* 2018). In our experiments, the median Rg of 10-min pulse labeled foci was ~30 nm (Fig. S8c), which is close to the lateral resolution of STORM imaging (~25 nm). As the spatial resolution (directly related with the single-molecule localization precision) sets the minimal apparent size of a target imaged by STORM, there would be no difference in the apparent size or position when imaging a 20 kb genomic region (10-min EdU labeling) and a much smaller sub-region (labeling for shorter than 10 minutes), e.g., the replication initiation site in the region. In addition, our STORM imaging showed that Rg of TADs is about 150 nm (Fig. 3e) and Rg of PCNA foci in the early S phase is about 100 nm (Fig. 4e). With 25 nm spatial resolution, STORM imaging is able to not only resolve individual 10-min labeled EdU foci (Rg~30 nm) but also map their distribution relative to TADs and PCNA foci. For SIM, it was equipped with an NA=1.40 oil objective and would provide ~120 nm spatial resolution used in the study by Belmont. While it is sufficient for imaging TADs and PCNA foci, this spatial resolution may not resolve multiple, individual replication foci within a TAD or near a PCNA focus.

2) *Cell synchronization methods using drug (thymidine followed by aphidicolin etc) may not be the best methodology to use. Aphidicolin may cause replication stress and it can alter chromatin architecture (as also acknowledged by the authors). The best synchronization procedures include 'cell elutriation' techniques.*

**Author reply:**

We thank the reviewer for raising the concerns on using drug to synchronize cells. Practically, as replication initiation takes place in a very short time window, we synchronized cells to the G1/S boundary to increase the probability for finding replication initiation events under the microscope. To eliminate the possibility that the thymidine/aphidicolin treatment may artificially lead to our observations, we designed new experiments to observe replication initiation sites at early S phase in cells without cell cycle synchronization. To do so, we applied BrdU labeling (green) for 1 hour, followed by 1 hour EdU labeling (red) and fixation of living cells cultured in a petri dish. As BrdU and EdU can only be globally incorporated into DNA during S phase, this strategy allows to identify the cell cycle stage of a cell based on its fluorescence color (Fig. R1a). For cells fixed in early S phase (cells with only red fluorescence), we refer to typical patterns of replication foci reported by our team (Q. Su … Y. Sun. *PNAS* 2020) and other teams (V. Chagin … M. Cardoso, *Nature Comm*, 2016) to select cells that had entered and been EdU-labeled for nearly 15 minutes like the purple punctate patterns shown in Figure R1b. By labeling TADs with FISH, we were able to measure the distribution of replication initiation sites relative to the TAD in non-synchronized cells. We proved that replication initiates at the periphery of a TAD in non-synchronized cells (Fig. R1b), consistent with the observation in aphidicolin-treated cells (Fig. R1b&c and Fig. 1a). Besides HeLa cells, we also checked other cell lines including hRPE, U2OS and MDA-MB-231 and obtained the same results (Fig. R1b&c). In summary, these new experiments conducted on non-synchronized cells helped to validate the conclusions obtained on synchronized cells.

Figure R1. **a**, schematic illustration of the strategy for labeling and distinguishing non-synchronized cells in early S phase; **b&c**, Super-resolution imaging of replication initiation sites and TADs in early S phase in synchronized or non-synchronized cells of different cell lines; P values show that spatial distribution of replication initiation sites in non-synchronized cells were close to that in aphidicolin synchronized cells as shown in Fig.1. P values were obtained by un-paired two sample parametric *t* test.

3) *The authors describe that the replication foci (based on the 10 min pulse, that they presume includes an origin) is at the periphery in TAD1 (early replicating domain) based on 10 and 15 min pulse experiments.  There is no control for 10-15 minutes pulse for TAD2* *(late replicating), only 60 mins is shown.*

**Author reply:**

We thank the reviewer for the question on TAD2. In this work, we focus on how chromatin structure regulates replication initiation in early S phase. Therefore, we performed 10 min or 15 min pulse labeling right upon release at the G1/S boundary. As TAD2 is a mid-late replication domain (Fig. 2a), we did not observe any replication foci for 60 min pulse labeling upon release at the G1/S boundary (Fig. 1), thus there would not be replication initiation in 10 min or 15 min time duration at the beginning of the S phase. Meanwhile, as cells lose synchrony gradually as soon as they are released at the G1/S boundary, it is difficult to label replication initiation events in TAD2 by 10 min pulse labeling in mid-S phase. Therefore, we chose to label replication origins in TAD2 by FISH at G1/S boundary and mid-S phase (Fig. 2b).

4) *All the conclusions of the manuscript are based on one TAD that is an early replicating domain and another one that is late replicating. Is this true globally? At least three examples need to be shown, each for early domain and late domain. Also, all the conclusions are drawn from a single cell line HeLa. It would be valuable to understand if similar organization is valid in different cell types, especially the non-cancerous ones.*

**Author reply:**

We thank the reviewer for raising the concerns on the generality of our conclusions based on one TAD and one cell line. In this revised manuscript, we have performed new experiments on 3 additional TADs, including 2 early replicating TADs (TAD3 and TAD4) and a late replicating TAD (TAD5). We have also performed new experiments on 3 additional cell lines, including hRPE, U2OS and MDA-MB-231. The results obtained on new TADs and new cell lines (Fig. S7, Fig. S12 and Fig. R1b&c) are consistent with that obtained on TAD1 and TAD2 in HeLa cells. In summary, these new experiments conducted on additional TADs and cell lines helped to validate the conclusions obtained in the original manuscript.

Figure R2. **Spatial distribution of replication origins in TAD3 TAD4 & TAD5 in the G1 and G1/S phases. a,** Representative STORM images of TADs (green, including TAD3, TAD4 and TAD5) and their origins (purple) labeled by FISH with oligoprobes in the G1 and G1/S phases. Top: TADs and active origins labeled at the G1/S transition. Middle: TADs and origins labeled at approximately 5 hours into the G1 phase. Bottom: TADs and dormant origins labeled at the G1/S transition. Portions of the two signals that overlap are shown in white. **b,** Barycenter distances between origins and TADs in **a** (n ≥15 cells). For lines and statistics in **b** see the description in the legend of Figure 1.

5) *The data do not support the conclusion that 'We obtained a global view of the spatial distribution of replication initiation sites relative to early RDs in a cell. Analysis of the dual-color STORM images showed that there were averagely 7 replication initiation sites in each RD.*

**Author reply:**

We thank the reviewer for raising the concerns on the spatial distribution of replication initiation sites relative to early RDs. We labeled early RDs by 45-min BrdU pulse upon release at the G1/S boundary in the first cell cycle. In the second cell cycle, we labeled replication initiation sites by 10-min EdU pulse labeling. We used SR-Tesseler segmentation algorithm to extract individual early RDs and replication initiation sites. The number of replication initiation sites labeled by EdU was 5132±672 and the number of early replicating domains labeled by BrdU was 732±185. Based on these analyses, we concluded that there were averagely 7 replication initiation sties in each RD. To be more precise, in the revised manuscript, we changed the sentence to “*We obtained a global view of the spatial distribution of replication initiation sites relative to early RDs that have replicated for 45-min upon release from the G1/S arrest”*.

6) *The correlation of different ORIs (based on Orc1 and H2AZ ChIP data) and TAD positioning was convincing and a strong data in support of the author's conclusions. It would be nice to use other examples to confirm that this holds true genome-wide.*

**Author reply:**

We are grateful for the positive comment. We also thank the reviewer for the suggestion. Based on the finding that H2A.Z marks active replication origins in our recently published article (H. Long…G. li, *Nature*, 2020), we chose active replication origins by combining Orc1 and H2A.Z ChIP data. There are thousands of TADs and tens of thousands of replication origins genome wide. High-throughput sequencing techniques cannot resolve the spatial distribution of replication origins within individual TADs. While STORM imaging is able to precisely map the intra-TAD origin distribution, it is difficult to genome-widely label TADs and origins with genomic specificity. To break through this limitation, we developed a high-throughput labeling strategy based on metabolic labeling of all early replicating TADs and their associated replication initiation sites by two rounds of BrdU and EdU pulse labeling at the beginning of the S phase over two consecutive cell cycles (Fig. 1c). We obtained a global view of the spatial distribution of replication initiation sites relative to early replicating TADs in a cell. Analysis of the dual-color STORM images showed that the spatially separated replication origins tend to initiate at the periphery of the early replicating TADs.

7) *For figure 3, where CTCF and Cohesin siRNAs have been performed, it would be good to have a control (For example, condensin siRNA).*

**Author reply:**

We thank the reviewer for the suggestion. In the work, we used a scramble RNA as a NC control because siRNA of a gene, such as *condensin*, may cause some disturbance to cellular physiology, such as cell cycle or chromatin structure organization (Fig. 3a&b and Fig. S13a&b).

8) *The authors note: 'Taken together, these data revealed that the structural re-organization within the TADs and decompaction in the G1 phase facilitate the relocation of highly-efficient origins from the TAD interior to the periphery'. What is the advantage of initiating at the periphery? It would be good to include that in the discussion*

**Author reply:**

We thank the reviewer for the inspiring question. We speculate 3 possible advantages for replication initiating at the periphery of a TAD.

(1) It could spatially separate the two key nuclear processes – transcription and replication, and thus help to prevent transcript-replication conflicts. Encountering between transcription and replication machineries is a major intrinsic source of genome instability. Our data suggested that the replication machineries are confined around the TAD and spatially separated from the transcription machineries, which mainly function within the TAD. Therefore, our work provides a new mechanism for cells to avoid the conflicts between replication and transcription based on spatial/topological separation. This point was included in the Discussion part of “Replication, transcription, and chromatin structure”.

(2) It has been reported recently (F. Ochs…C. Lukas, *Nature*, 2019) that “53BP1 and RIF1 form an autonomous functional module that stabilizes three-dimensional chromatin topology at sites of DNA breakage. This process is initiated by accumulation of 53BP1 at regions of compact chromatin that colocalize with TAD, followed by recruitment of RIF1 to the boundaries between such domains.”. The key role of TAD borders in the maintenance of genome architecture and stability for DNA repair suggests a possible mechanism that DNA replication and repair coordinate at the TAD periphery.

(3) Previous studies have shown that PCNA clusters in the nucleus are of constrained mobility. Replication origins at the TAD periphery may be more accessible to the replication machineries. This point was included in the Discussion part of “The Chromatin Re-organization Induced Selective Initiation (CRISI) Model”: “*That the chromatin structural dynamics within the TADs make origins accessible to immobile PCNA clusters provides an interesting viewpoint to understand protein-DNA interactions in the nucleus, which are commonly considered to be based on diffusive search of proteins such as transcription factors on chromatin DNA. Such mechanisms may be involved in various nuclear functions. For example, during DNA damage repair, ATM is restricted at the double strand breaking (DSB) site while phosphorylation of H2AX by ATM spreads over a domain. The discrepancy between the distribution of the kinase (ATM) and its product (γH2AX) can be explained by the local movements of the chromatin fiber inside the TAD which bring distant nucleosomes to spatial proximity of ATM.*” These possibilities would be interesting subjects for future studies.

9) *'Upon down-regulation of CTCF or cohesin using RNAi (Figure 3a insets, Figure 3b, left panel), we found that the highly efficient origins (ORI2 and ORI3) were not relocated to the TAD periphery at the G1/S transition in both 2D and 3D images.' Did this lead to change in the timing of DNA replication? What was the consequence?*

**Author reply:**

We thank the reviewer for the inspiring questions. David Gilbert has recently reported that “CTCF depletion had almost no effect on replication timing genome wide” (J. Sima…D. Gilbert, *Cell*, 2019). Combining this observation of replication timing and ours on replication origin selection, it is suggested that disruption of TAD structure by CTCF/cohesin depletion would scramble the selection of active origins. Therefore, sub-TAD chromatin structural re-organization is particularly essential for differential origin selection in TADs. In addition, another study showed that cohesin is required for chromatin loop formation and depletion of cohesin decreases the number of active origins in a TAD (E. Guillou…J. Mendez, *Genes Dev*, 2010). This observation is in consistent with our findings.

10) *A recent study (as noted above) pointed that 'early constitutive origins fire most frequently within or adjacent to TAD boundaries genome-wide'. And it was proposed: 'active transcription emanating from CTCF binding sites displaced replication initiation sites towards the edges of the TAD boundary.' The observations made by the current paper also support the view that relocation of replication origins is dependent on chromatin-looping, however there are key differences that need clarification.*

**Author reply:**

We thank the reviewer for pointing out the work by Lombardi and Tarsounas, and we have cited it in the discussion part of our revised manuscript. Lombardi and Tarsounas (E. P. Lombardi and M. Tarsounas, *bioRxiv*, 2020) reported that 'early constitutive origins fire most frequently within or adjacent to TAD boundaries genome-wide'. This sequencing-based finding is consistent with our imaging-based finding as the sequence boundary of a TAD corresponds to its physical boundary (B. Bintu… X.W. Zhuang, *Science,* 2018). However, this work cannot explain the active replication origins between the TAD boundaries. Using super-resolution imaging, we found that origins inside the TAD which could be relocated to the TAD periphery are also highly active. Based on spatial replication initiation distributions rather than sequencing patterns, our data reveal that spatial location of the origins in the domain is more important than their linear location for origin activation.

11) *It is known that without cohesin, mammalian TAD-like domains can form spontaneously by chromatin interactions. Does this impact the replication within these kinds of TADs?*

**Author reply:**

We thank the reviewer for the nice question. The formation mechanism of TAD itself is an open question. Previous studies have suggested that TADs can be formed in manners both CTCF/cohesin dependent and independent. In this work, we only disrupted CTCF/cohesin-dependent TADs and demonstrated their roles in regulating replication origin activation. Intriguingly, some studies have shown that most CTCF/cohesin-independent TADs are mediated by enhancer-promoter interactions, and our transcription interference experiments also lead to changes in replication origin selection, implicating that origin activation may also be regulated by chromatin structure in CTCF/cohesin-independent TADs. This assumption would be an interesting subject to study in the future.

12) *The transcription inhibition experiments are interesting, but do not address some concerns. For example, inhibiting transcription in the fly has been shown to cause reorganization of TAD structure, and a weakening of border strength. Further, Cavalli's group has shown that transcription by itself is insufficient and that transcription factors may insulate CTCF-independent TAD borders. Also, using STORM imaging, Cavalli group demonstrated that within TADs active domains were found at the borders of repressed ones. The authors in this manuscript suggest, 'that transcription-dependent chromatin structural re-organization within the TADs exposes a subset of origins to the physical boundary of a TAD, which are preferentially used for replication initiation.' Recent work shows that when* *transcription is inhibited chemically or deregulated by oncogene overexpression, replication origins become repositioned to the centre of the TAD. It may be important to discuss these studies in the current manuscript and discuss the caveats of the transcription inhibition experiments.*

**Author reply:**

We thank the reviewer for pointing out these papers regarding the relationship between TAD and transcription. Transcription inhibition in Drosophila revealed that the structure and boundaries of TAD would be affected (L. Li…V. G. Corces, *Mol Cell.*, 2015). However, because the size and other characteristics of TAD differ between Drosophila and humans, it is not very appropriate to apply the findings in Drosophila to humans. As for the Cavali’s experiment (B. Bonev…G. Cavalli, *Cell*, 2017), they used sequencing techniques and found that transcription is not sufficient to influence TAD boundary. However, our super-resolution imaging experiment focused on the intra-TAD spatial structural changes caused by transcription, which cannot be resolved by sequencing. As for the study that transcription inhibition caused by oncogene overexpression is related with replication origin relocalization towards TAD center (E. P. Lombardi … M. Tarsounas, *bioRxiv*, 2020), it is consistent with our finding that transcription inhibition abolishes relocation of active replication origins to the TAD periphery. We have included discussion on pertinent points in our revised manuscript.

13) *The data using MCM and PCNA is not convincing. PCNA is found in soluble nucleoplasmic pool and a preextraction procedure is critical to look at the chromatin-associated form. No such methodology has been used (as per details provided in the methods section). Further, the authors conclude that PCNA foci/clusters at the TAD boundary is the reason why origins at TAD periphery get activated. Detailed experiments are needed to support this claim.*

**Author reply:**

We thank the reviewer for raising the concerns on the soluble portion of PCNA in the nucleus. Because pre-extraction procedure based on high salt treatments is likely to disrupt chromatin structure, we instead performed mild extraction using 0.5% triton. In addition, during STORM data analysis, we applied a clustering threshold to eliminate dispersed, soluble PCNA molecules and retain PCNA clusters. These PCNA clusters are bound to chromatin because others’ (X. Deng …A.S. Belmont, *Current Biology*, 2016) and our previous work (Q. P. Su…Y. Sun, *PNAS*, 2020), both found that PCNA clusters are highly colocalized with RFi at the beginning of the S phase.

Importantly, the observation that PCNA clusters remain around TADs from the G1 to the beginning of the S phase suggests that the location of PCNA cluster is not determined by DNA replication. We thus proposed that “*This spatial distribution of PCNA provides a plausible reason why origins at the TAD periphery get preferentially initiated.*” in the original manuscript. As it is difficult to obtain direct experimental evidence, which is also beyond the scope of the current study, we chose to tone down the statement to “*This spatial distribution of PCNA clusters provides a possible explanation for preferential initiation of origins at the TAD periphery.”* in the revised manuscript.

14) *Data suffers from overinterpretation: 'The CRISI model suggests that the spatial localization of an origin in a TAD determines its replication efficiency.' There are no experiments supporting efficiency of replication.*

**Author reply:**

We thank the reviewer for the question on the efficiency of replication. For DNA replication, the replication efficiency is defined as “the probability of origin firing”. Therefore, high replication efficiency refers to active origins and low replication efficiency refers to dormant origins. As we observed that origins with different spatial localizations have different probabilities of firing, the CRISI model suggests that the spatial localization of an origin in a TAD determines its replication efficiency.

**Reviewer #3:**

*This manuscript presented a study that revealed a new mechanism for selecting replication origin using two-color STORM imaging to map the spatial distribution of replication origins in the context of topologically associating domains (TAD). They labeled two specific TAD sites (one enriched in early replicating domain and one enriched in mid-replicating domain) and used pulse labeling of EdU and BrdU to compare the spatial co-localization between these two TAD sites and replication origins in G1 and G1/S phase. They reported that replication origins initiated separately at the periphery of a TAD. They found that chromatin reorganization within the TADs in G1 phase, which is driven by disruption of chromatin loops during transcription elongation. The overall studies are rigorous and thorough. The findings are interesting to biologists in the field of DNA replication and genome organization. I recommend the publication of this manuscript after minor revision.*

**Author reply:**

We are very grateful to the reviewer for the overall positive comment on our work.

1) *The authors used BrdU and EdU labeling for either replication origins or replication domains.* *BrdU is based on antibody labeling, which often suffers from much lower labeling density compared to click chemistry-based labeling. How did the authors ensure the unbiased comparison?*

**Author reply:**

We thank the reviewer for raising the professional question on the labeling density of BrdU. As for the BrdU labeling density, we agree that it is indeed lower than EdU. To investigate the influence from low labeling density, we calculated the Rg and mass center position of a TAD containing a typical number of localizations (2547) for simulation. We randomly took out half or three quarters of localizations to investigate how labeling density affects Rg and mass center position. The mean value and standard deviation of Rg were as follows: All points (mean 160.3 nm); Half points (mean:160.2 nm std:1.5 nm); Quarter points (mean:159.6 nm, std: 2.2 nm). The mean value and standard deviation of mass center position were as follows: All points (mean (1937.9 nm, 1881.8 nm)); Half points (mean: (1937.4 nm, 1882.3 nm) std: (1.3 nm, 2.5 nm)); Quarter points (mean: (1937.0 nm, 1881.7 nm), std: (2.7 nm, 4.6 nm)). Each calculation was under the statistics from 30 tests. The results indicate that labeling density has minimal influence on the calculation of barycenter distance.

2) *In Methods, the identification of barycenter is a crucial factor in this study, but* *how the barycenter is defined here was not clearly described. As barycenter involves the calculation of cluster mass, how was "mass" defined in STORM images was not clear.*

**Author reply:**

We apologize for the confusion on the description of barycenter and “mass” in the previous manuscript. We utilized the pointillist nature of STORM data for calculation. Thus, each localization for calculation was treated as a point with “mass” of 1 unit. In this way, the barycenter of a TAD or a replication site was calculated as the mass center of all single molecule localizations it contains. We have updated this part in the revised manuscript.

3) *In line 424 in Supplementary Materials, the authors stated "Different density factors were evaluated for identification of TADs and small foci such as protein clusters or replication origins". This description was too vague.*

**Author reply:**

We thank the reviewer for raising the concerns on density factor. Density factor was used in the SR-Tesseler software as a threshold for object and cluster segmentation, which was based on the average molecule density δ defined in the article (F. Levet … J. B. Sibarita. *Nat Methods* 2015):

To make the description of “density factor” more clearly, our statement in the methods has been changed to “Different density factors were evaluated for identification of TADs and small foci such as protein clusters or replication origins (Fig. S4). For analyzing FISH or metabolically labeled TADs, density factor was set to 3. For analyzing FISH or metabolically labeled replication origins or initiation sites, density factor was set to 20. For analyzing protein clusters, density factor was set to 3.5.” in the revised manuscript. Parameter optimization of the density factor is described in detail in Figure S4:

“Origins close to each other in a TAD could not be separated when the density factor was set from 2 to 10. Origins were too small or even missing when the density factor was set to 30. When the density factor was set to 20, approximately 5,000 origins were clearly identified at the beginning of the S phase in one cell, similar with a previous report (V. O. Chagin…M. C. Cardoso, *Nat Comm*, 2016). c, TADs close to each other could not be separated when the density factor was set to 2. TADs were too small or even missing when the density factor was set from 5 to 30. When the density factor was set to 3, approximately 700 TADs were clearly identified, similar with a previous report (D. A. Jackson, A. Pombo, *J Cell Biol*, 1998). Therefore, density factor was set to 3 for analyzing TADs and 20 for analyzing RFi”.

**2^nd^ round**

**Reviewer 2**

The authors have answered most of my concerns satisfactorily. A. couple of minor points that need to be addressed include the following:

1) Fig S12: 'Bottom: TADs and dormant origins labeled at the G1/S transition.

Portions of the two signals that overlap are shown in white.' Was aphidicolin used in these experiments? If so, the use of aphidicolin can result in the activation of dormant origins and can result in changes to the localization.

2) Please remove the term 'lowly efficient origins' and replace with origins that fire with low efficiency. "Lowly' would have a different meaning.

3) Fig. S6: This is a clever strategy, but more details need to be provided in the methods section. One hour of BrdU labeling, is the BrdU degraded after one hour (and what concentration was used to ensure that all is incorporated)? It is clear that the early and mid/late S-phase can be distinguished using this protocol, but. Unclear how the authors can ascertain G2/M phase. These cells are not being monitored in live.

4) The following statement is still unclear 'This spatial distribution of PCNA clusters provides a possible explanation for preferential initiation of origins at the TAD periphery.' PCNA is chromatin-bound only in S-phase. I am not convinced that there is chromatin-bound PCNA during G1 considering that no pre-extraction procedure was used. Even fluorescently tagged PCNA shows homogenous localization during G1. The authors do correctly point out that work from others showed that PCNA clusters are highly colocalized with RFi at the beginning of the S phase.

5) Details of the statistical analyses needs to be included for each figure.

**Reviewer 3**

The authors fully addressed my previous comments. I now recommend it for publication.

**Authors’ response**

1) *The authors have answered most of my concerns satisfactorily. A. couple of minor points that need to be addressed include the following:*

**Author reply:**

We thank the reviewer for his/her encouraging evaluation of our revised manuscript. We are also grateful for the additional comments and suggestions.

2) *Fig S12: 'Bottom: TADs and dormant origins labeled at the G1/S transition. Portions of the two signals that overlap are shown in white.' Was aphidicolin used in these experiments? If so, the use of aphidicolin can result in the activation of dormant origins and can result in changes to the localization.*

**Author reply:**

We thank the reviewer for this question. We did use aphidicolin in the experiments in Fig. S12. In order to avoid selection of dormant origins abnormally activated by aphidicolin treatment, we combined two separate replication profiles with and without aphidicolin treatment and picked to label dormant origins that are not activated under aphidicolin treatment. The first replication profile is the dynamic replication profile measured by BrdU-seq under aphidicolin treatment. The second replication profile is the replication landscape measured by OK-seq without aphidicolin treatment.”

3) *Please remove the term 'lowly efficient origins' and replace with origins that fire with low efficiency. "Lowly' would have a different meaning.*

**Author reply:**

We thank the reviewer for the suggestion and we have changed the statement in the manuscript.

4) *Fig. S6: This is a clever strategy, but more details need to be provided in the methods section. One hour of BrdU labeling, is the BrdU degraded after one hour (and what concentration was used to ensure that all is incorporated)? It is clear that the early and mid/late S-phase can be distinguished using this protocol, but. Unclear how the authors can ascertain G2/M phase. These cells are not being monitored in live.*

**Author reply:**

We thank the reviewer for his/her appreciation of our strategy to address the aphidicolin concern in the revised manuscript. In this protocol, the concentration of BrdU is over-saturated and not degraded. We applied 3 rounds of PBS buffer to wash out the small BrdU molecules.

We also thank the reviewer for his/her concern on “G2/M phase” identification. We checked Fig. S6 and noticed that “G2/M” in the second row is a typo. It is supposed to be “G2” when observing “green followed by blank”. For “G2/M”, as the duration from the end of S phase to G2/M phase is about 4 hours for HeLa cells, there would no observable fluorescence signal for either BrdU or EdU labeling. We have now corrected the typo in this revision.

5) *The following statement is still unclear 'This spatial distribution of PCNA clusters provides a possible explanation for preferential initiation of origins at the TAD periphery.' PCNA is chromatin-bound only in S-phase. I am not convinced that there is chromatin-bound PCNA during G1 considering that no pre-extraction procedure was used. Even fluorescently tagged PCNA shows homogenous localization during G1. The authors do correctly point out that work from others showed that PCNA clusters are highly colocalized with RFi at the beginning of the S phase.*

**Author reply:**

We thank the reviewer for the question about PCNA, particular in the G1 phase. In the paper by Heilemann’s group (Scientific Reports | 6:18779 | DOI: 10.1038/srep18779), They used single-particle tracking to investigate the dynamics of PCNA in live mammalian nuclei in both S phase and non S-phase. They observed two distinguishable modes of PCNA mobility, one slow and one fast population for both stages (S and non S-phase) of the cell cycle (Fig. 2A). In non-S cells, they observed 15 ± 8% (s.e.m.) of PCNA molecules in a slower population with a diffusion coefficient and concluded that a small fraction of PCNA in non S-phase nuclei exists that is involved in complex formation or binding to structures independent of DNA replication.

In our work, we did not investigate whether PCNA bound to chromatin in G1 phase in Figure 4c. In the revised manuscript, we had included the following discussion: “Regarding the constrained mobility of PCNA clusters in the nucleus, we speculate three possible mechanisms that are not mutually exclusive. Firstly, PCNA and the replisomes are giant complexes binding DNA with low diffusive mobility. Secondly, PCNA and the replisomes may be attached to the nuclear matrix (Figure 5c), which is supported by an immunoelectron microscopy study showing that DNA polymerase ɑ, PCNA, and nascent DNA are colocalized in nucleoskeleton bodies [69]. Thirdly, the proteins comprising replisome complexes might form liquid condensates that phase-separate from TADs [70]. These possibilities would be interesting subjects for future studies.”

6) *Details of the statistical analyses needs to be included for each figure.*

**Author reply:**

We thank the reviewer for the suggestion. We have included the details of the statistical analyses.
